# Recent trends in scientific research in chest radiology: What to do or not to do? That is the critical question in research

**DOI:** 10.1007/s11604-025-01735-3

**Published:** 2025-01-16

**Authors:** Hiroto Hatabu, Masahiro Yanagawa, Yoshitake Yamada, Takuya Hino, Yuzo Yamasaki, Akinori Hata, Daiju Ueda, Yusei Nakamura, Yoshiyuki Ozawa, Masahiro Jinzaki, Yoshiharu Ohno

**Affiliations:** 1 Department of Radiology, Center for Pulmonary Functional Imaging, Brigham and Women’s Hospital and Harvard Medical School, 75 Francis St., Boston, MA 02115 USA; 2 Diagnostic and Interventional Radiology, Osaka University Graduate School of Medicine, Osaka, Japan; 3 Department of Radiology, Keio University School of Medicine, Tokyo, Japan; 4 Department of Clinical Radiology, Graduate School of Medical Sciences, Kyushu University, Fukuoka, Japan; 5 Department of Artificial Intelligence, Graduate School of Medicine, Osaka Metropolitan University, Osaka, Japan; 6 Department of Diagnostic Radiology, Fujita Health University School of Medicine, Toyoake, Aichi Japan; 7 Joint Research Laboratory of Advanced Medical Imaging, Fujita Health University School of Medicine, Toyoake, Aichi Japan

**Keywords:** Chest radiology, CT, Photon-counting CT, Upright CT, Dynamic chest radiography, Magnetic resonance imaging

## Abstract

Hereby inviting young rising stars in chest radiology in Japan for contributing what they are working currently, we would like to show the potentials and directions of the near future research trends in the research field. I will provide a reflection on my own research topics. At the end, we also would like to discuss on how to choose the themes and topics of research: What to do or not to do? We strongly believe it will stimulate and help investigators in the field.

## Introduction

The snow started falling on the forest in New England, while I am working on this “honorable” invited review manuscript for “Insights from leading experts” collection. As a serious physician investigator, “how to choose research topics” has been always in the center of my mind for myself and for over 100 young fellows and trainees I worked with. It is often underestimated that “What not to do” is as important as “What to do”, because any small projects may occupy your mind at least for 3–6 months, sometimes 1–2 years. In the current review, I invited the rising young investigators in chest radiology from all over Japan to demonstrate the potentials and directions of the near future research trends in chest radiology: Dr. Yanagawa and Dr. Hata from Osaka University, Dr. Yamada from Keio University, Dr. Hino and Dr. Yamasaki from Kyushu University, Dr. Ueda from Osaka Metropolitan University, and Dr. Ozawa from Fujita Health University for “MR of the lung”. I added my own reflection and discussion “how to choose research topics”. Dr. Nakamura, currently at Brigham and Women’s Hospital, helped the overall organization.

I wish this article will stimulate and help young investigators in the field and may give some clues when they think of what research subjects they choose.

## High-spatial-resolution CT and photon-counting CT in thorax

### High-spatial-resolution CT

The spatial resolution of CT in energy-integrating detector CT (EID-CT) has been in the range of 0.23 to 0.35 mm in-plane and from 0.50 to 0.625 mm through-plane [1, 2]. However, the advancement of spatial resolution technology in EID-CT has enabled the implementation of high-spatial-resolution (HSR) CT since 2017, achieving spatial resolutions of up to 140 µm in-plane and 250 µm through-plane [3]. HSR CT can enhance spatial resolution, reduce streak and dark band artifacts, and deliver superior image quality compared to conventional EID-CT, resulting in highly detailed information regarding lung anatomy and disease states. As for the field of view (FOV), HSR CT can show improved image quality even when the FOV is reduced to about 80 mm, as its resolution exceeds the pixel size. While noise in HSR CT increases with reduced FOV, it can remain comparable to conventional EID-CT at FOVs of 240 mm or larger, where clinical impact is minimal [4]. Moreover, compared to conventional CT scanners, 1024 × 1024 or a 2048 × 2048 matrix can be used on HSR CT. When the FOV is set to 320 mm to cover the entire lung, the theoretical pixel size is 0.625 mm for a 512 × 512 matrix, 0.313 mm for 1024 × 1024, and 0.156 mm for 2048 × 2048. In HSR CT scans, a larger matrix size maintains spatial resolution, improves image quality, and enhances lung disease assessment, despite increased noise compared to a 512 matrix size [5].

From a clinical perspective, the interruption of the air bronchogram, characterized by a sudden discontinuity in bronchioles with a diameter of ≥ 0.5 mm on HSR CT, was a significant imaging biomarker of the invasiveness in lung adenocarcinoma [6]. Two radiomic features, coefficient of variation and entropy, derived from HSR CT with a 1024 matrix, were significant predictors of solid and micropapillary components in lung invasive adenocarcinoma which were known to have worse survival prognosis and higher recurrence rates [7]. Diffuse pulmonary ossification (DPO) is common in fibrosing interstitial lung disease (ILD), particularly in idiopathic pulmonary fibrosis (IPF), and its CT signs may aid in diagnosing IPF [8]. Nodular pulmonary ossifications are present in over 80% of patients with honeycombing on HSR CT, where they are more easily detectable than with conventional CT, supporting ILD diagnosis [9]. The enhanced spatial resolution of HSR CT may subjectively and objectively help to elucidate various pathologies, such as interstitial lung abnormalities, interstitial lung disease, smoking-related lung injury, and lung cancer, potentially enabling more accurate prognosis prediction and the formulation of tailored treatment plans for each patient.

**Fig. 1:**
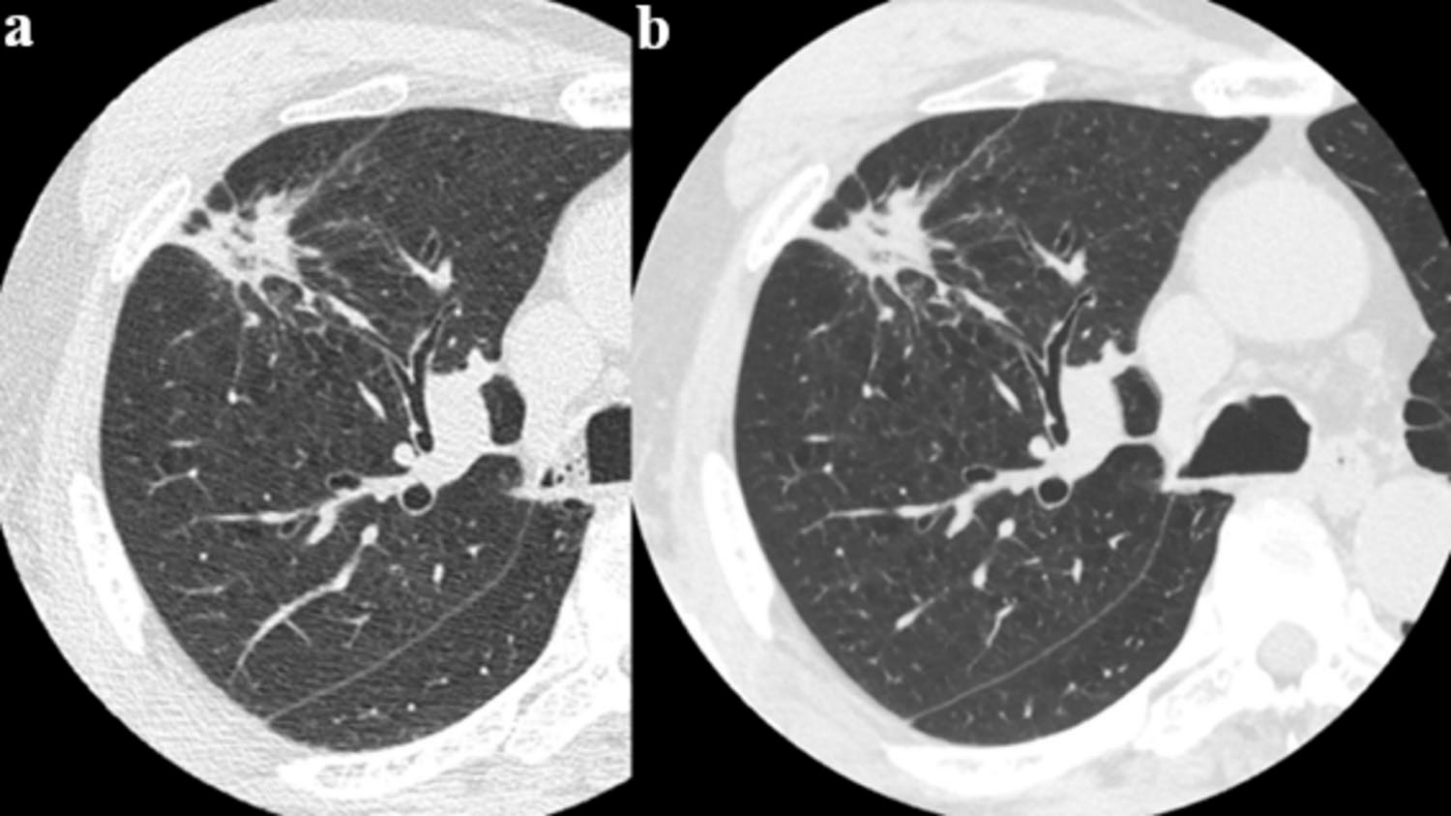
**a** Filtered-back projection image with 512 matrix and 0.5 mm slice thickness. **b** Precise IQ Engine (PIQE) image with 1024 matrix and 0.5 mm slice thickness derived from **a**. PIQE image provided high spatial resolution with no artifacts and even less noise

**Fig. 2:**
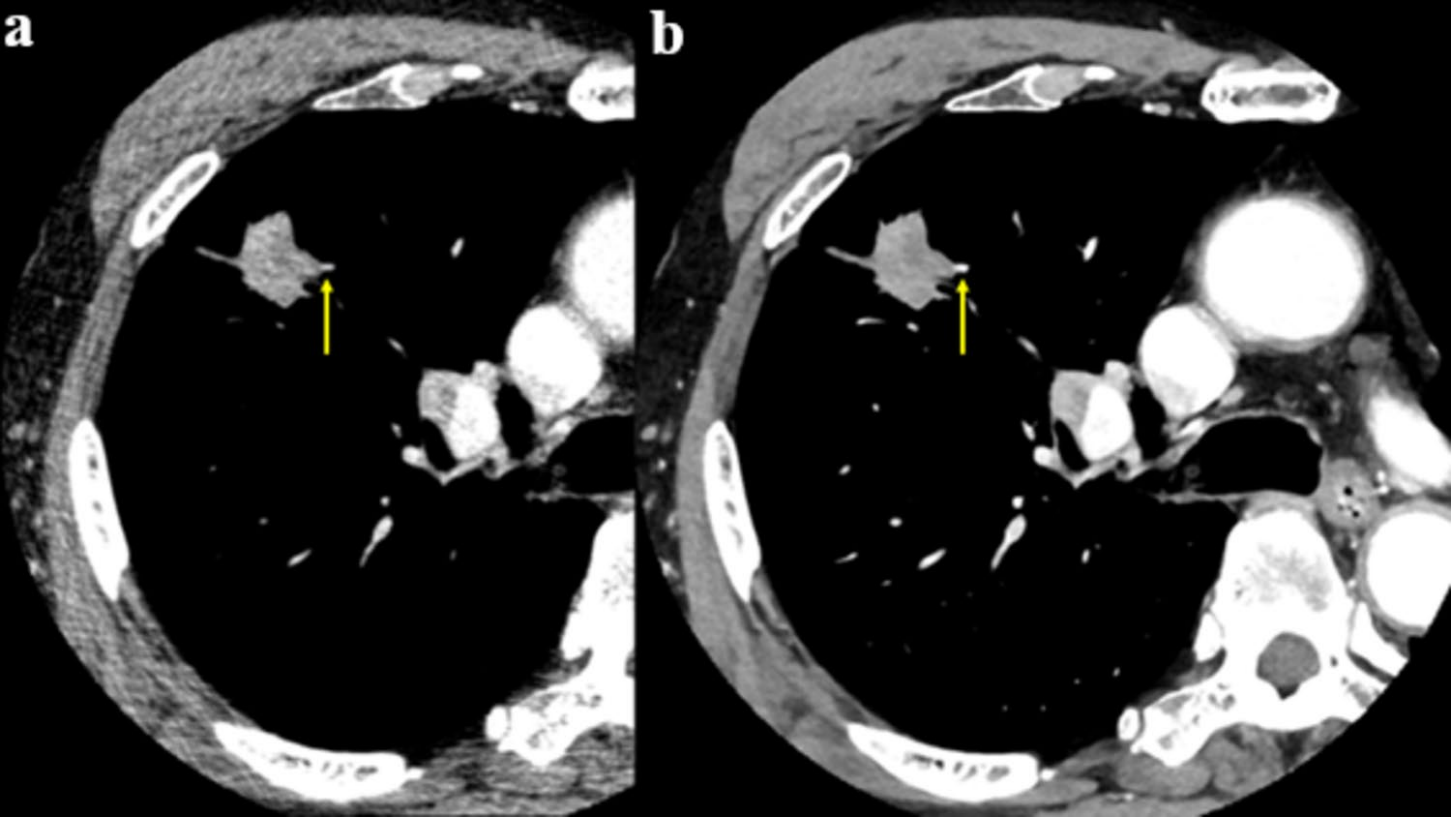
**a** Filtered-back projection image with 512 matrix and 0.5 mm slice thickness. **b** Precise IQ Engine (PIQE) image with 1024 matrix and 0.5 mm slice thickness derived from **a**. PIQE image provided high spatial resolution with no artifacts and even less noise. Small blood vessels can be detected more clearly (arrows)

Artificial intelligence (AI)-based techniques, including deep-learning-based reconstruction (DLR), are increasingly applied in diagnostic imaging [10, 11]. In particular, super-resolution DLR (Precise IQ Engine [PIQE], Canon Medical Systems, Japan) reduces noise and enhances spatial resolution by reconstructing low-noise, high-resolution images using a deep convolutional neural network trained on HSR CT data [12–14]. Even with conventional EID-CT, the application of PIQE reconstruction technology can enhance image resolution (Figs. 1 and 2), which not only improves image quality but is also expected to impact diagnostic performance in the future.

### Photon-counting CT

**Fig. 3:**
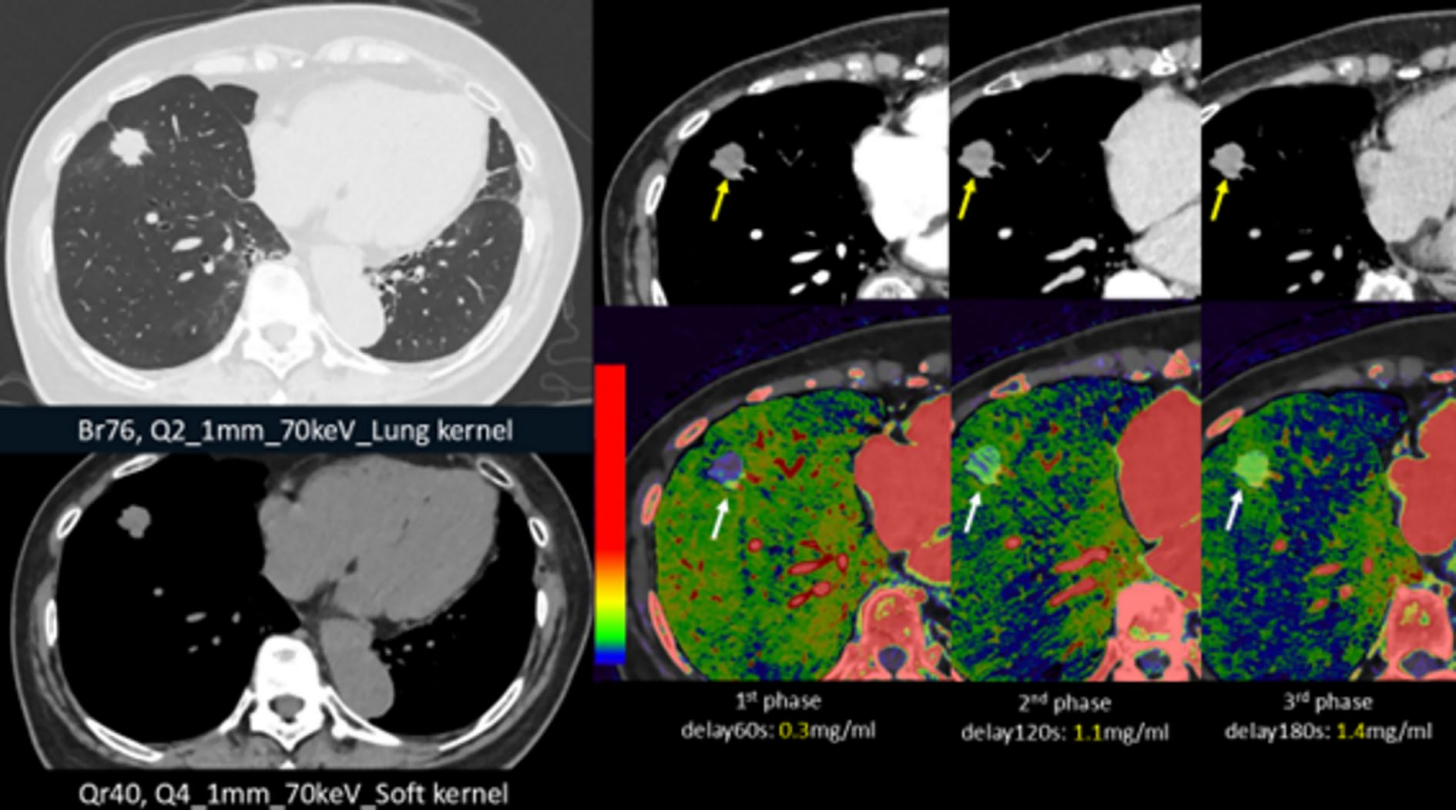
PC-CT can provide high spatial resolution and precise iodine mapping at the same time. Utilizing PC-CT in oncology has the potential to enhance lung cancer staging, therapy planning, and treatment response assessment, as well as detecting incidental pulmonary nodules by improving subjective and objective evaluations

Photon-counting CT (PC-CT) surpasses EID-CT by offering high-resolution and spectral imaging capabilities at lower radiation doses and reduced contrast agent usage. PC-CT can provide spatial resolutions of up to 110 µm in-plane and 200 µm through-plane [15–17]. PC-CT has demonstrated superior performance in detecting and evaluating small nodules and airways compared to EID-CT [17]. The high spatial resolution achieved through a large matrix size, thin-slice thickness, and small FOV further enhanced detection capabilities. This technology has the potential to identify submillimeter nodules and airways with greater precision. PC-CT can also produce the creation of virtual monochromatic, virtual non-contrast (VNC), and material decomposition images such as precise iodine mapping, which enhances both visual and quantitative evaluations (Fig. 3) [18].

In the future, the high feature stability of PC-CT supports radiomics and AI applications, while advancements like k-edge imaging may enable simultaneous imaging of multiple agents [19, 20], enhancing diagnostic potential and early cancer detection. Moreover, PC-CT contributes to green radiology by offering higher resolution while reducing radiation dose and the use of iodinated contrast agents, aligning with environmental sustainability goals.

## Upright CT

**Fig. 4:**
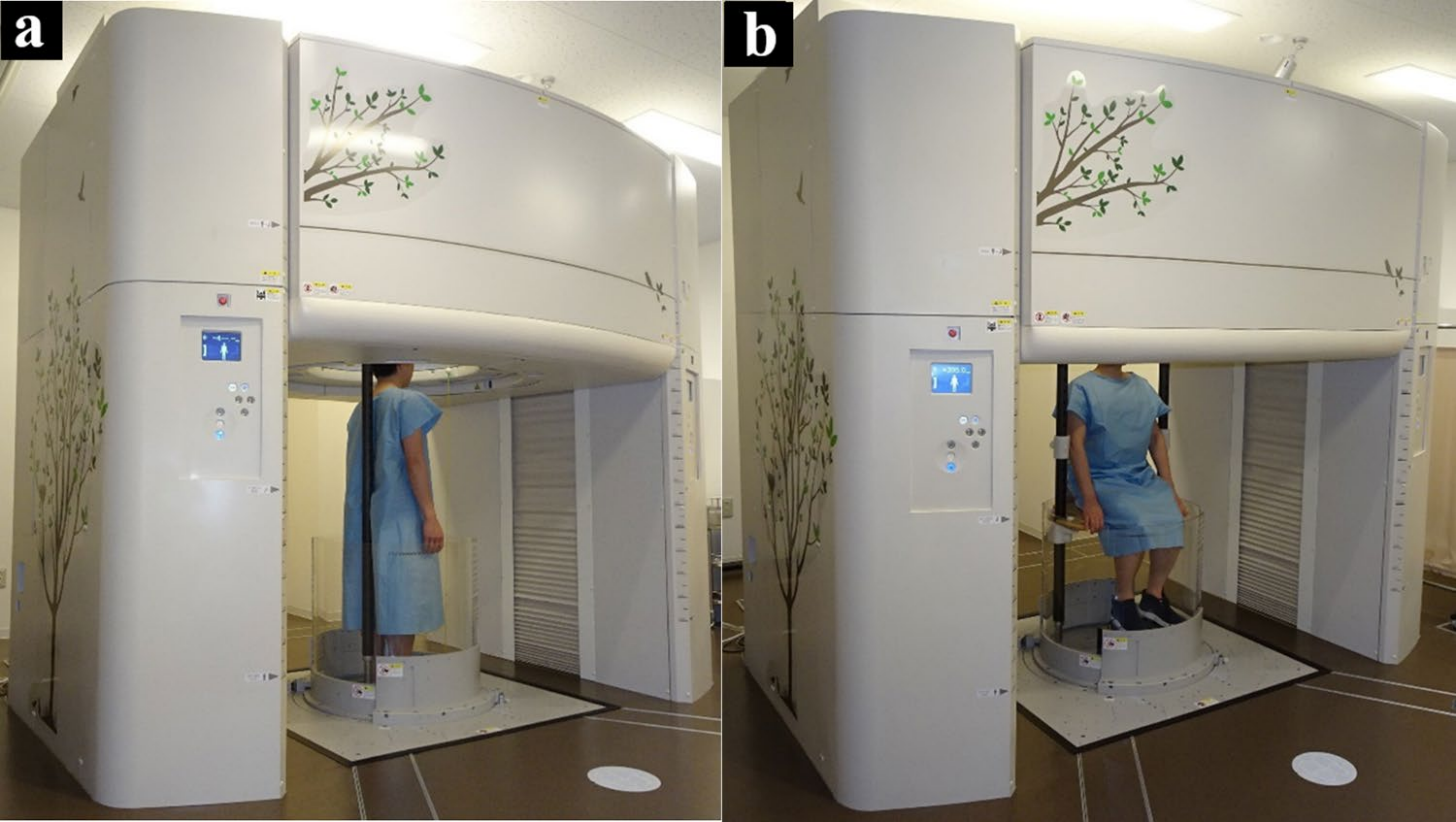
Upright CT examinations performed in the standing (**a)** and sitting (**b)** positions. The upright CT system features up-and-down movement of a transverse 320-detector-row gantry. For enhanced safety during upright scanning, an acrylic enclosure surrounds the subject to prevent falls, equipped with both a pinch prevention and contact interlock mechanism. To provide stability for standing subjects (**a)**, a 2.3-m-long carbon back-support pole is installed, extending from the floor to the top of the system. This pole’s position is adjustable according to the subject’s body dimensions or specific scan requirements. In addition, for elderly or frail patients, a Velcro band can be attached to the pole and gently secured around the patient for support. As this subject is a healthy volunteer, the Velcro band is not applied

Humans spend most of their day in an upright (standing or sitting) position, yet most 3-dimensional diagnostic imaging modalities, such as MRI and CT, are conducted in a supine position. Although cone-beam CT can acquire cross-sectional images in an upright position, it provides limited soft tissue information due to low contrast, and its narrow bore size restricts the scan range [21]. To address these limitations, Dr. Jinzaki and his colleagues, in collaboration with Canon Medical Systems (formerly Toshiba Medical Systems), proposed and developed an upright CT scanner capable of whole-body imaging (Fig. 4) [22]. Phantom studies have demonstrated that the noise characteristics, spatial resolution, and CT values of upright CT are comparable to those of the supine 320-row-detector CT, confirming its diagnostic reliability [22].

**Fig. 5:**
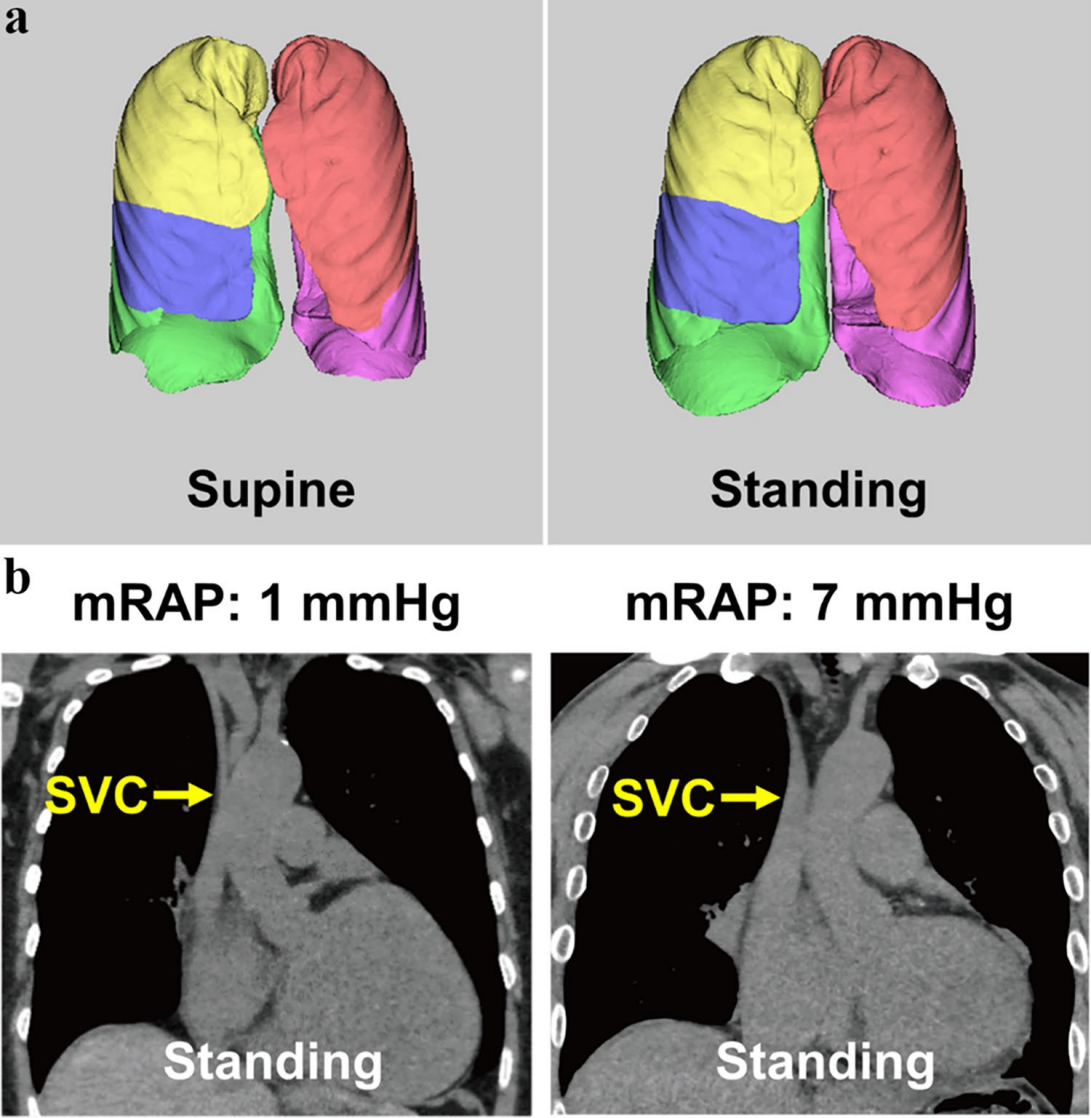
Volume-rendering supine and upright CT images illustrating lung/lobe volume measurements in a 51-year-old man (**a)**, as well as coronal upright CT images from a patient with a mean right atrial pressure (mRAP) of 1 mmHg and another patient with mRAP of 7 mmHg **(b)**. **a** The right upper lobe is shown in yellow, the right middle lobe in blue, the right lower lobe in green, the left upper lobe in pink, and the left lower lobe in purple. **b** The diameter of the superior vena cava (SVC, indicated by arrows) in the patient with a mRAP of 7 mmHg (right) is larger than that in the patient with a mRAP of 1 mmHg (left)

Because upright CT is performed with the patient standing, total access time—from entering to starting imaging and from imaging completion to exit—is less than half that of conventional supine CT, thereby improving workflow efficiency [22]. Studies using both supine and upright CT imaging in healthy volunteers and patients have shown that gravity induces anatomical changes throughout the body—affecting the brain, lungs/lobes (Fig. 5a), airways, heart, pelvic floor, spine, joints, and systemic veins—when transitioning from supine to upright positions [22–30]. In a cohort of healthy volunteers, the inspiratory and expiratory bilateral upper and lower lobe and lung volumes, as well as the volumes and luminal areas of the airways, were reported to be significantly higher in the standing/sitting positions than in the supine position (Table 1) [25, 31], with total lung volume increasing by 9%/10% in inspiratory standing/sitting positions and 6.4%/9.5% in expiratory standing/sitting positions. However, the inspiratory right middle lobe volume remained similar across all three positions, while the expiratory right middle lobe volume was significantly lower in the standing/sitting positions (16.3%/14.1% decrease) compared to the supine position (Table 1) [25]. In studies with healthy individuals and patients with chronic obstructive pulmonary disease (COPD) or idiopathic pulmonary fibrosis, lung volume and airway measurements on upright CT showed stronger correlations with pulmonary function indices, such as total lung capacity, vital capacity, functional residual capacity, residual volume, and forced expiratory volume in one second [23, 25, 31–34]. Therefore, upright CT may be useful for predicting certain pulmonary function test outcomes in clinical settings. Furthermore, upright CT provides an enhanced physiological assessment of cardiovascular structures. A recent study showed that upright CT measurements of the superior vena cava for assessing elevated right atrial pressure (> 5 mmHg) demonstrated an area under the curve (AUC) of 0.91 (95% confidence interval [CI], 0.77–1.00), which was superior to the AUC of 0.78 (95% CI, 0.59–0.98) observed with supine CT (Fig. 5b) [35]. This suggests that upright CT may have a future role in non-invasive cardiovascular evaluations, particularly in assessing patients with fluctuating or elevated venous pressures. As of November 2024, upright CT is available in only five facilities in Japan. However, given its ability to provide novel findings that reflect patient physiology under daily conditions, upright CT is expected to expand worldwide in the coming years.

**Table 1:** The average percent increase/decrease of change in lung, lobe, and airway among the supine, sitting, and standing positions (100 healthy volunteers)

	Average percent increase/decrease in volume in standing position compared to that in supine position	Average percent increase/decrease in volume in sitting position compared to that in supine position	Average percent increase/decrease in volume in sitting position compared to that in standing position
Total (bilateral) lung volume (inspiratory)	+ 9.2%	+ 10.0%	+ 0.7%
Right lung volume (inspiratory)	+ 8.5%	+ 9.7%	+ 1.1%
Right upper lobe volume (inspiratory)	+ 7.9%	+ 8.5%	+ 0.6%
Right middle lobe volume (inspiratory)	− 0.1%	+ 0.9%	+ 1.1%
Right lower lobe volume (inspiratory)	+ 12.0%	+ 13.6%	+ 1.4%
Left lung volume (inspiratory)	+ 10.1%	+ 10.3%	+ 0.2%
Left upper lobe volume (inspiratory)	+ 6.4%	+ 6.8%	+ 0.4%
Left lower lobe volume (inspiratory)	+ 13.8%	+ 13.8%	− 0.0%
Total (bilateral) lung volume (expiratory)	+ 6.4%	+ 9.5%	+ 2.9%
Right lung volume (expiratory)	+ 5.3%	+ 8.7%	+ 3.2%
Right upper lobe volume (expiratory)	+ 12.2%	+ 13.8%	+ 1.4%
Right middle lobe volume (expiratory)	− 16.3%	− 14.1%	+ 2.5%
Right lower lobe volume (expiratory)	+ 9.1%	+ 14.6%	+ 5.0%
Left lung volume (expiratory)	+ 7.6%	+ 10.4%	+ 2.6%
Left upper lobe volume (expiratory)	+ 5.9%	+ 7.1%	+ 1.1%
Left lower lobe volume (expiratory)	+ 9.9%	+ 14.7%	+ 4.4%
Airway volume (inspiratory)	+ 2.5%	+ 4.6%	+ 2.0%
Airway volume (expiratory)	+ 13.4%	+ 14.9%	+ 1.4%
Luminal area of the trachea (inspiratory)	+ 4.6%	+ 4.2%	− 0.4%
Luminal area of the right main bronchus (inspiratory)	+ 6.1%	+ 7.1%	+ 0.9%
Luminal area of the left main bronchus (inspiratory)	+ 9.3%	+ 8.9%	− 0.4%
Luminal area of the average third-generation airway (inspiratory)	+ 7.8%	+ 10.3%	+ 2.4%
Luminal area of the trachea (expiratory)	+ 6.6%	+ 6.4%	− 0.2%
Luminal area of the right main bronchus (expiratory)	+ 9.9%	+ 12.0%	+ 1.9%
Luminal area of the left main bronchus (expiratory)	+ 11.8%	+ 12.8%	+ 0.9%
Luminal area of the average third-generation airway (expiratory)	+ 8.0%	+ 10.2%	+ 2.1%

## Dynamic X-ray for diaphragm and lung

DCR (dynamic chest radiography) is a technique of sequential X-ray irradiation [36]. DCR system consists of a flat paned detector and X-ray pulse generator with higher efficiency. DCR constantly generates X-ray with high temporal resolution as high as 15 frames per second, and offers sequential images [36, 37]. Radiation exposure by 15-s examination can be reduced to from 0.3 to 1.0 mGy, which is lower than ordinary chest radiograph [37].

DCR has several advantages toward CT or MRI. The procedure for DCR examination is as simple as that for ordinary chest radiograph; acquisition time is much shorter [37]. DCR also provides images easily in standing position as well as supine position. DCR images of bilateral or unilateral lung fields in particular respiratory phase can be obtained. Pixel value change of sequential images helps to identify the very phase of inspiration or expiration [38].

**Fig. 6:**
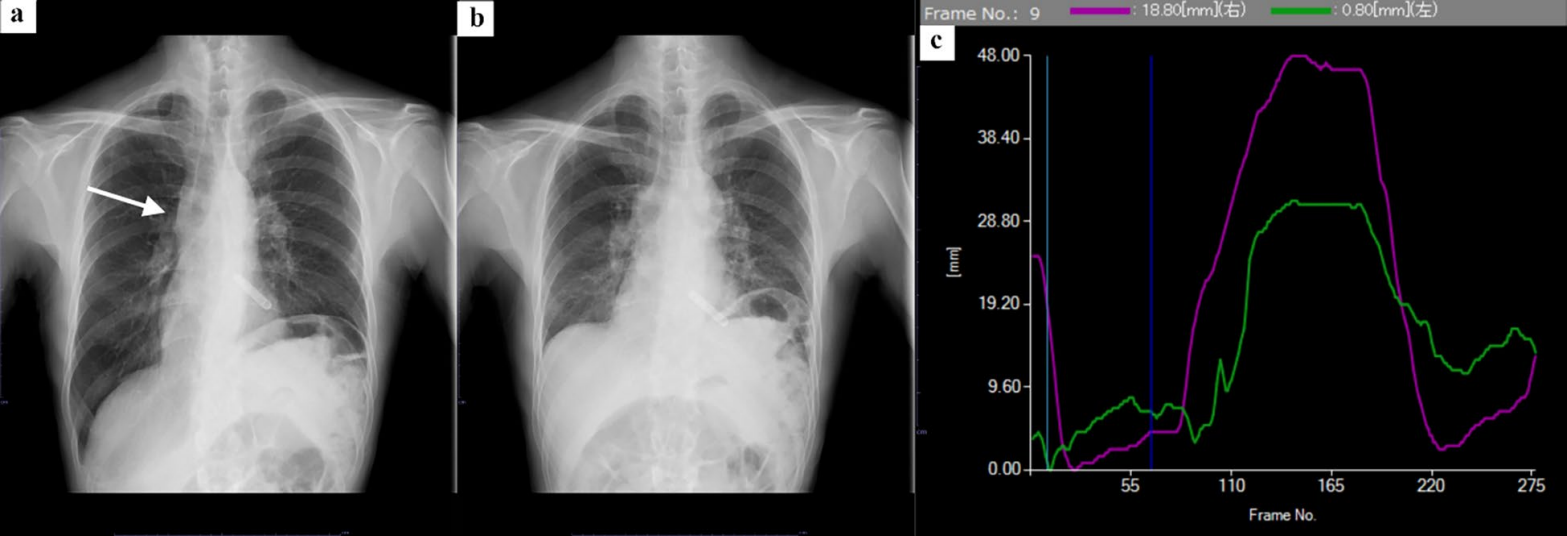
79-year-old man with thymic mass. **ab** Mediastinal mass (arrow) was suspected by chest X-way images in forced inspiration and forced expiration, which was confirmed as thymic cancer by percutaneous biopsy. **c** Temporal change of diaphragm excursion was visualized with curved graph. The motion of left diaphragm (green curve) is smaller than that of right diaphragm (purple curve), suggesting left phrenic nerve paralysis due to tumor invasion

**Fig. 7:**
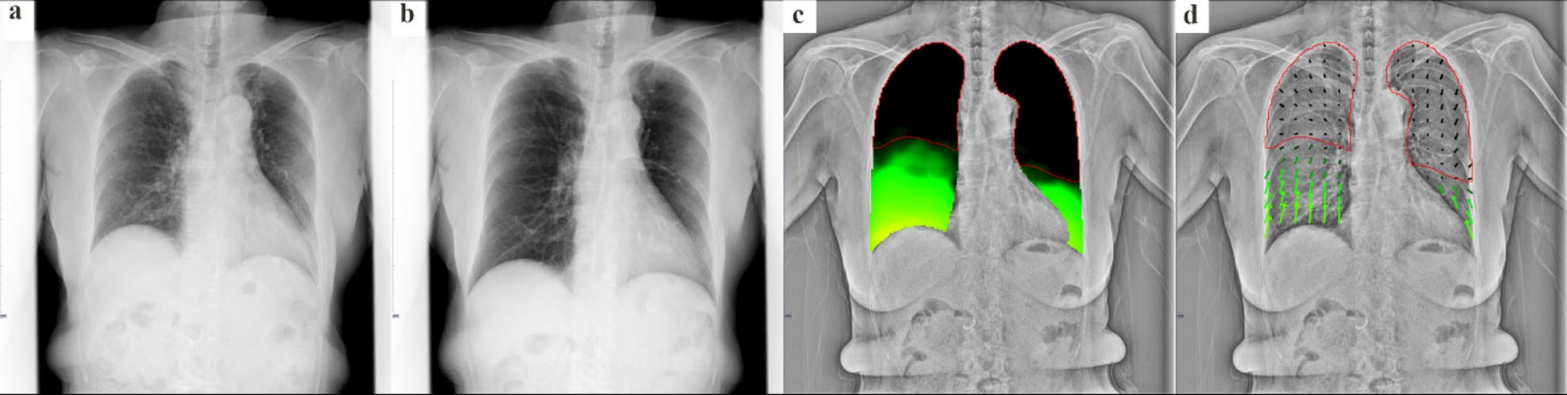
79-year-old female with past history of partial resection of left upper lobe. **ab** DCR images in forced inspiratory and expiratory phase, respectively. Nodular opacity was observed in the inner region of left inner-upper lung field, which was confirmed as adenocarcinoma by pathological examination. **c** Colored map and **d** Vector map. Concentration gradient of sequential images was converted to these maps with optical flow method to visualize motion of lung parenchyma. Area in left apex, circled with curved red line, was that with little change of pixel value, suggesting adhesion of lung to apex after lung surgery. Adhesion of left lung was visually recognized during surgery

The temporal change of anatomy or pixel value can be analyzed automatically by the workstation for the various studies or analyses. DCR is now applied for the evaluation of dynamic motion, ventilation, and perfusion by utilizing its high temporal resolution [38–47]. Previous studies have reported the excursion of diaphragm measured by DCR; the excursion of motion speed of diaphragm was different in patients with pulmonary obstructive pulmonary disease (COPD), restrictive lung disease cystic fibrosis, compared with normal subjects [39–45]. Dynamic tracheal narrowing could be observed with DDR, which was useful to measure the change of tracheal diameter precisely [46, 47]. Projected lung area is the area of lung fields excluding both mediastinum and heart, which showed moderate correlation with vital capacity in normal volunteers [38]. Projected lung area and its temporal change had the possibility to reflect on the pulmonary function [38, 43, 45, 47]. Lung field motion or inspiratory lung volume was also analyzed by the application of pixel value change [48–51]. Decreased lung motion by DCR was also applied to the evaluation of chest adhesion as preoperative assessment [52, 53]. Representative cases of chest DCR are shown in Figs. 6 and 7.

Portable system of DCR has been recently launched. Portable DCR is performed in anteroposterior direction, while ordinary DCR in posteroanterior direction. Hence, further studies are recommended for the optimization of collection and interpretation of image data [54].

## Dynamic X-ray for pulmonary perfusion

**Fig. 8:**
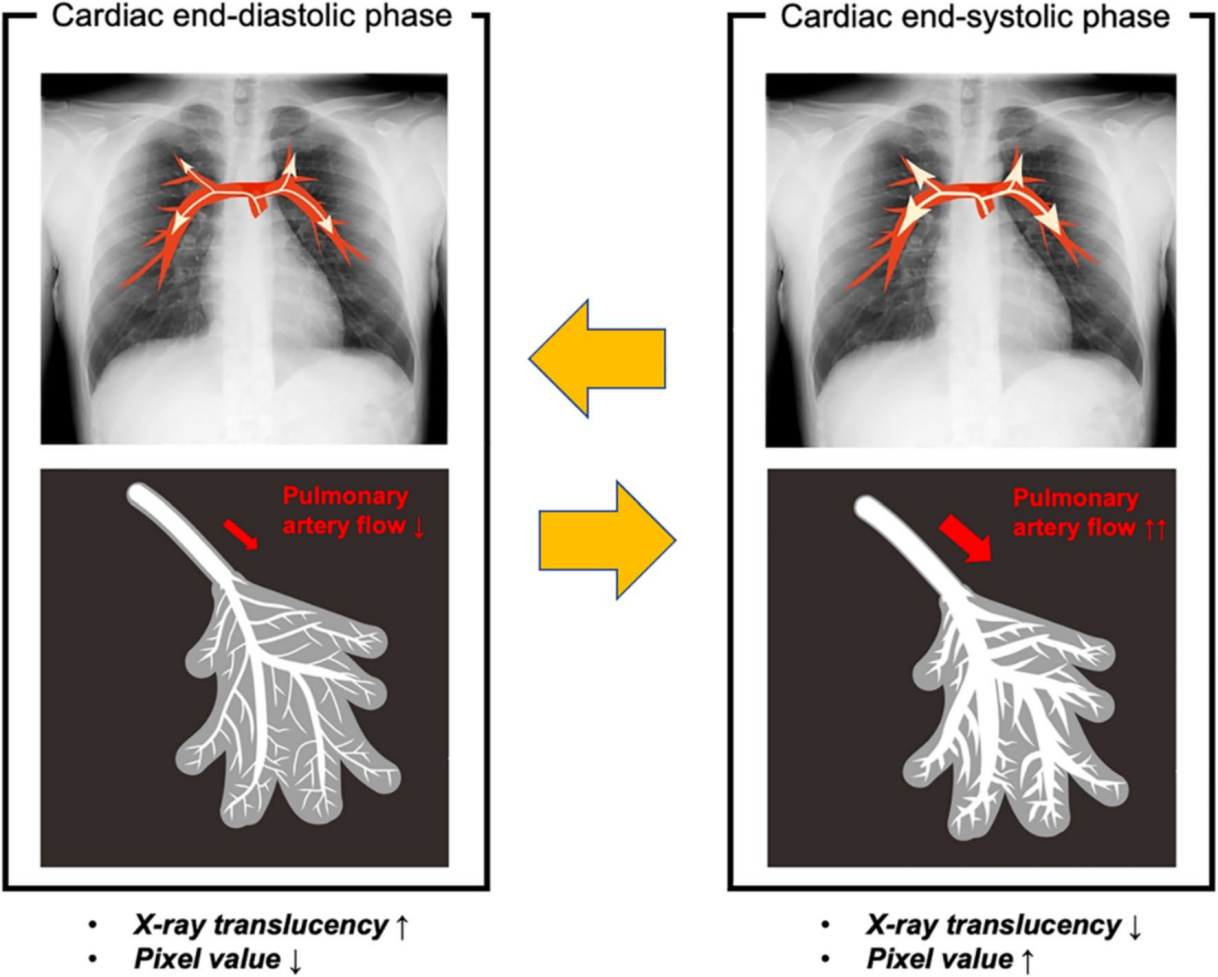
Theory of perfusion imaging of dynamic chest radiography. Pulmonary artery flow increases during the systolic phase, leading to increased blood and vessel volume in the lungs. This results in a slight temporal decrease in X-ray translucency. Dynamic chest radiography analyzes small temporal changes in X-ray translucency (pixel value) by cardiac pumping and visualizes the pulmonary perfusion. * This figure was adapted from Ref. 60 under the Creative Commons Attribution 4.0 International License

Lung perfusion evaluation using dynamic chest radiography (DCR) is based on the analysis of temporal changes in X-ray translucency of the pulmonary arteries [55]. Blood volumes in the pulmonary arteries in the systolic phase were greater than those in the diastolic phase because the heart pumps blood into the pulmonary arteries in the systolic phase (Fig. 8). These differences in sequential images during breath-holding are analyzed and visualized as lung perfusion in DCR. Therefore, this technique allows non-invasive evaluation of lung perfusion without contrast media or radionuclides.

Pulmonary embolism (PE) is a disease for which DCR is the most suitable. In a retrospective study, DCR demonstrated 66–70% sensitivity, 92–93% specificity, and 86–88% accuracy in detecting acute PE [56]. Importantly, DCR increased the diagnostic ability for PE detection irrespective of the observer’s expertise and experience. Regarding the detection of chronic thromboembolic pulmonary hypertension (CTEPH), the sensitivity, specificity, and accuracy of DCR are reported to be 97%, 86%, and 92%, respectively [57]. CTEPH is a type of pulmonary hypertension, meaning that most patients with CTEPH have extensive pulmonary perfusion defects in their lungs, contributing to a higher performance than in acute PE. With further technological advancements, including the integration of AI, DCR has the potential to become a more reliable tool for diagnosing PE. The simplicity of DCR has recently enabled the development of a portable system [54], potentially allowing for future installation in mobile units. This could offer an opportunity to diagnose PE in residents of remote areas that lack access to CT scans or lung scintigraphy [58, 59].

**Fig. 9:**
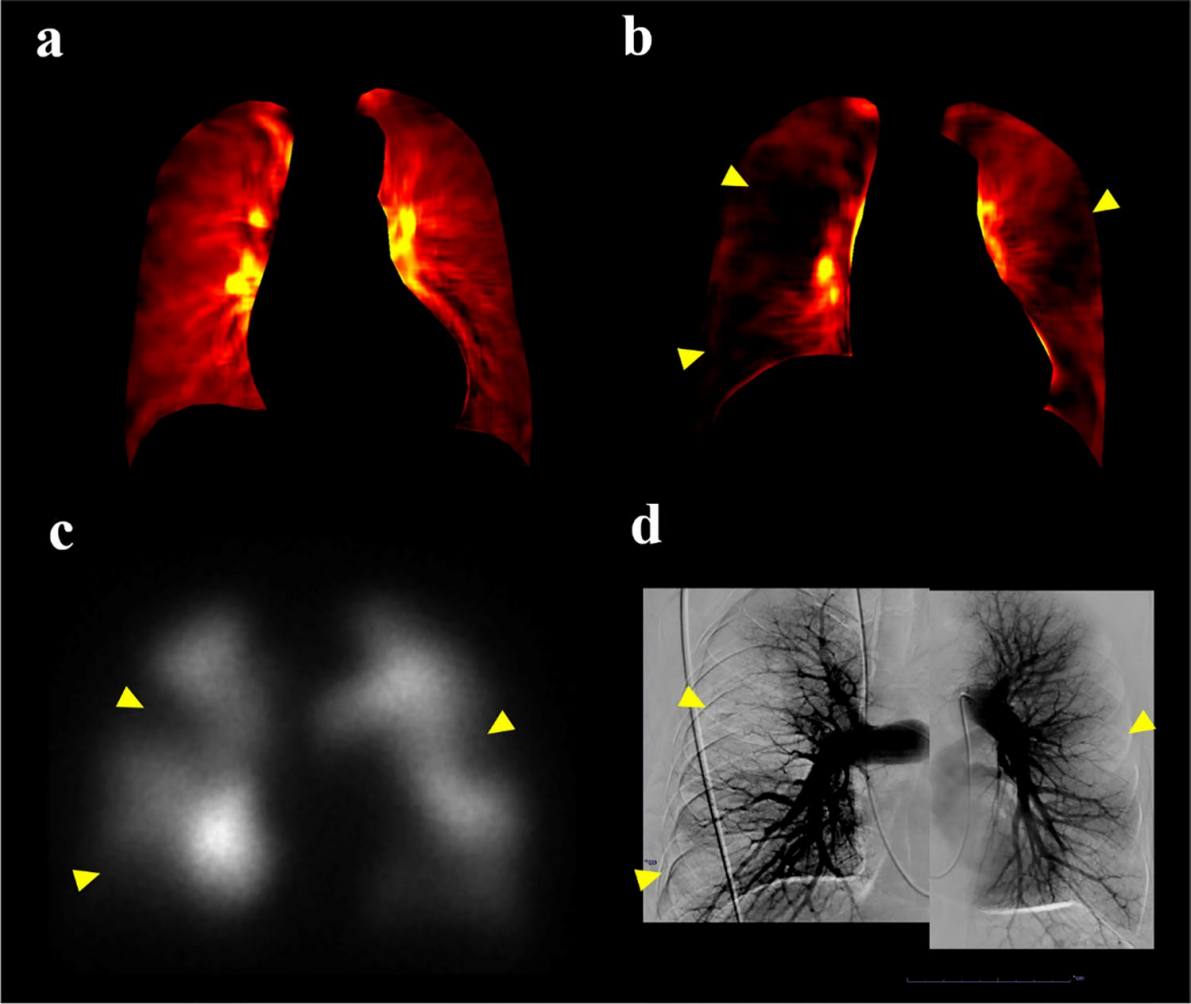
**a** Dynamic chest radiography image of a patient with pulmonary hypertension due to an etiology other than chronic thromboembolic pulmonary hypertension (CTEPH) showing uniform, clear perfusion in both lungs. **b** Dynamic chest radiography, **c** anterior planar perfusion scintigraphy, and **d** invasive pulmonary angiography images of a patient with CTEPH demonstrating multiple wedge-shaped perfusion defects in both lungs (arrowheads)

DCR imaging findings show a strong correlation with those of the anterior view in planar perfusion scintigraphy, the coronal view of the iodine map in CTPA, and invasive pulmonary angiography (Fig. 9) [60, 61]. DCR enables both visual and semi-quantitative evaluations of various pulmonary vascular diseases, presenting significant potential for clinical applications. Several cases of DCR demonstrating clinical utility have been reported in pulmonary vein stenosis, large vessel arteritis, adult congenital heart diseases, and pulmonary arteriovenous malformation [62–64]. DCR has also been reported to be useful in predicting hemodynamics measured by right heart catheterization and in the assessment of left heart failure, with further applications anticipated in the field of cardiology [65, 66]. Further research in larger cohorts is required to validate the reported findings and explore additional clinical utilities.

## AI for interstitial lung abnormalities

In recent years, considerable research has been conducted on the clinical significance of interstitial lung abnormalities (ILA) [67, 68]. In most studies, the presence of ILA is determined through visual assessment by radiologists or pulmonologists. However, inter-observer agreement among human evaluators is not perfect, with recent studies reporting inter-reader κ values ranging from 0.43 to 0.82 [69–71], highlighting variability in reproducibility. Efforts have also been made to develop automated methods for assessing ILA. This shift toward automation reflects the growing need for objective and standardized methods to overcome the limitations of visual interpretation, particularly in multicenter studies and large-scale clinical trials.

Chae et al. utilized deep learning-based texture analysis to evaluate ILA [72]. The AI system automatically segmented features such as normal lung parenchyma, emphysema, consolidation, ground-glass attenuation (GGA), reticular abnormalities, honeycombing, and/or traction bronchiectasis. By quantitatively assessing GGA, reticular abnormalities, and honeycombing as findings indicative of ILA, they reported a diagnostic performance with an area under the curve (AUC) of 0.99 when compared against human assessment as the ground truth. This segmentation-based quantification approach enables numerical representation of lesion intensity, which could be advantageous for monitoring disease progression over time. However, a drawback of this method is the loss of spatial information regarding lesion locations, as well as the difficulty in distinguishing ILA from other conditions that similarly cause GGA.

We have developed an AI model for automatic ILA identification using a different approach [73]. Our model combines two components: one AI system calculates the probability of ILA presence on a per-slice basis for CT images, while another aggregates these slice-based probabilities to determine the overall probability of ILA in the entire CT examination. This model enables automated identification of ILA from a single chest CT image series and may account for common distribution patterns of ILA, such as its tendency to appear in subpleural regions or at the lung bases. However, our approach does not necessarily capture the severity of abnormalities. The diagnostic performance of our model, with human evaluation as the ground truth, achieved an AUC of 0.87. It is important to recognize that human visual assessment is not entirely accurate. Therefore, further investigation is needed to determine the clinical utility of these AI models, particularly their ability to predict clinical outcomes, such as survival. These AI-based evaluations offer a highly reproducible means of assessing ILA, potentially aiding clinical decision-making and supporting future clinical trials.

## AI for chest radiography

Artificial intelligence (AI) applications in chest radiography have evolved rapidly since Lakhani et al.’s pioneering work on deep learning for tuberculosis diagnosis in 2017 [74]. This development has followed two distinct paths: augmenting existing diagnostic capabilities and enabling novel analytical approaches previously thought impossible.

The application of AI to existing diagnostic tasks has led to significant progress in lung cancer detection. A recent study showed that AI-based computer-aided detection (CAD) systems can achieve a sensitivity of 0.73 with 0.13 false positives per image for nodule detection [75]. Moreover, when such systems are integrated into clinical workflows, they significantly improve general physicians’ diagnostic performance, increasing sensitivity from 0.47 to 0.60 while maintaining high specificity (0.96–0.97) [76]. These improvements are particularly notable given that traditionally, 19–26% of visible lung cancers are missed during initial radiograph interpretation [77, 78].

**Fig. 10:**
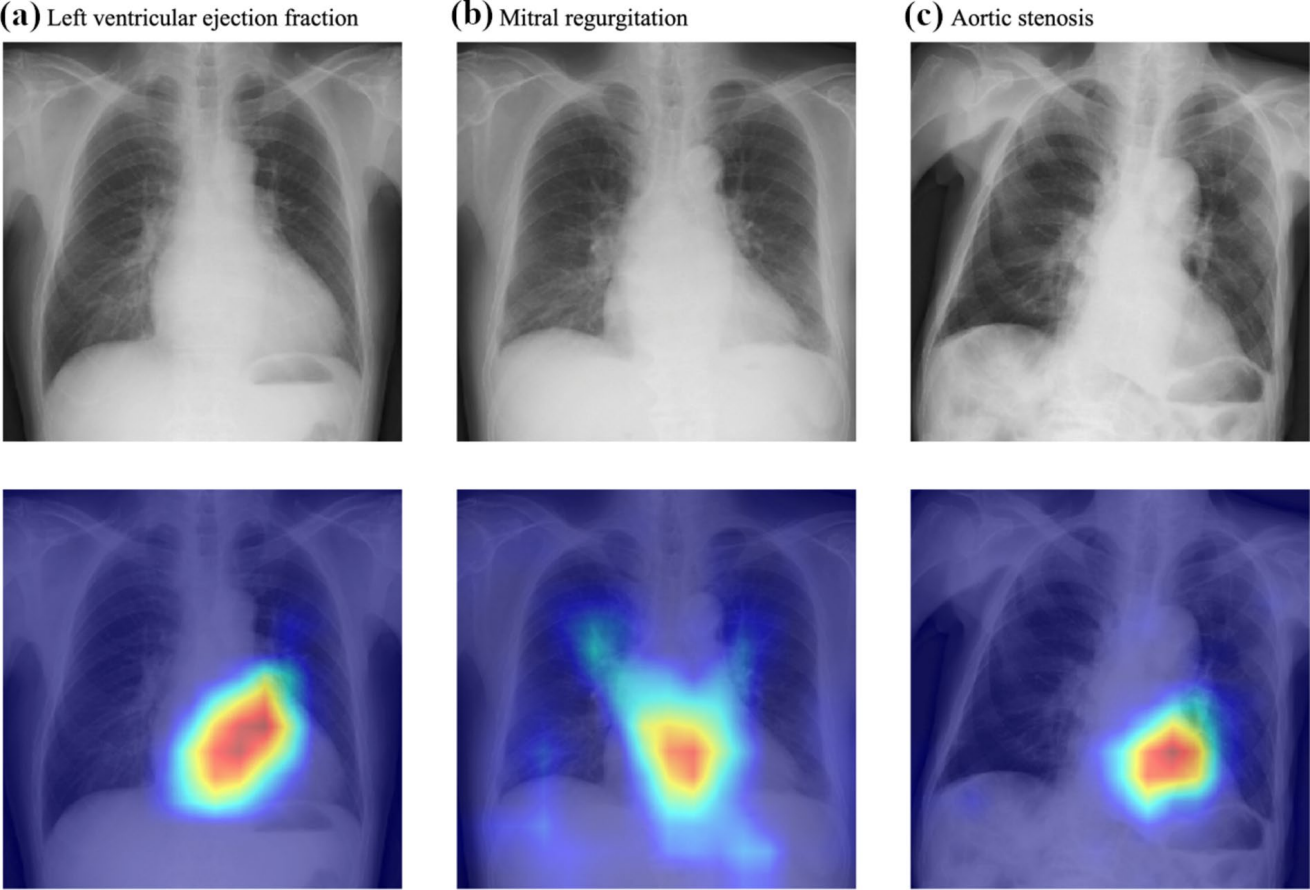
Chest radiographs (above) and corresponding saliency maps (below) demonstrating three different cardiac conditions. **a** Low left ventricular ejection fraction: the chest radiograph shows cardiomegaly. The saliency map highlights the cardiac short axis with high intensity (red–yellow). **b** Mitral regurgitation: The chest radiograph demonstrates an enlarged cardiac silhouette with prominent pulmonary vasculature. The saliency map shows intense activation from the central cardiac region to the hilar region. **c** Aortic stenosis: the chest radiograph shows cardiac enlargement with possible aortic valve calcification. The saliency map’s activation is focused in the aortic region, consistent with the pathology location

**Fig. 11:**
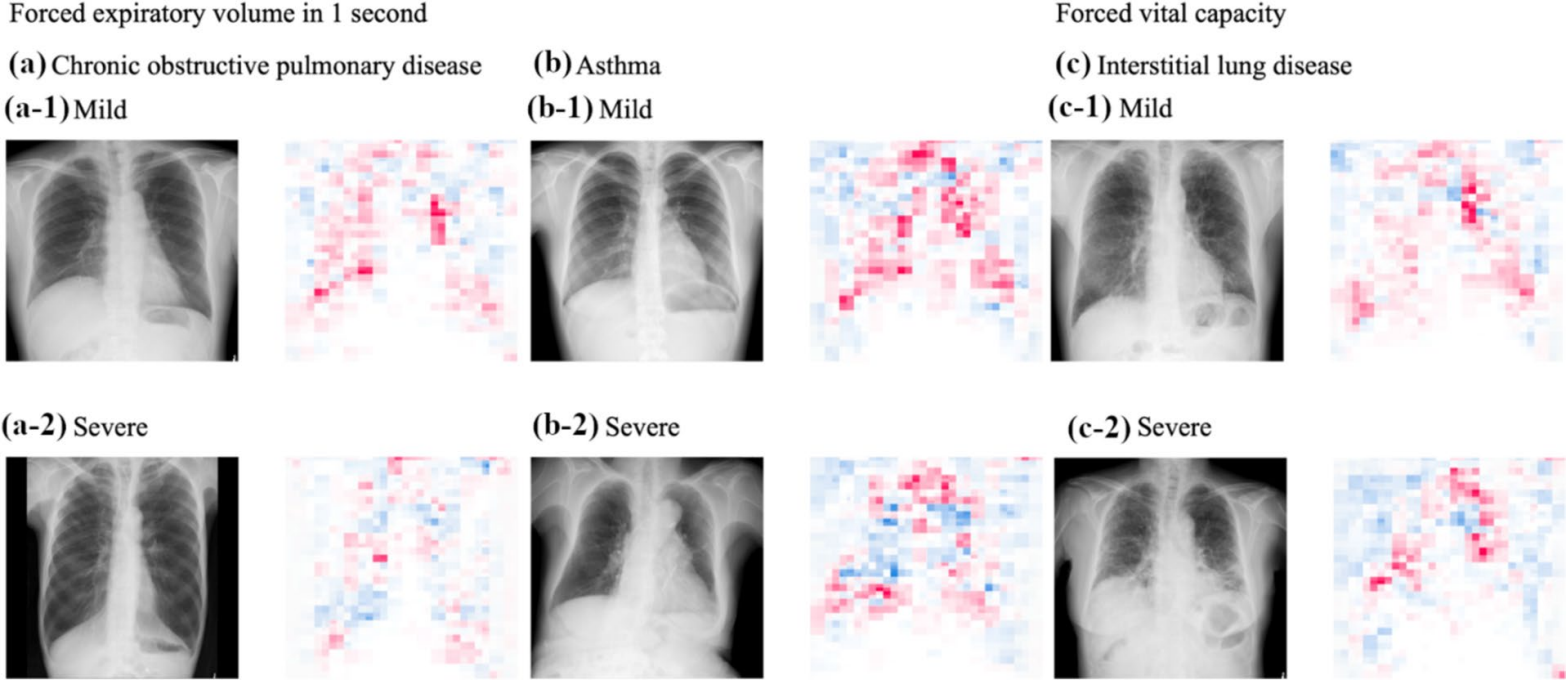
Chest radiographs and corresponding saliency maps for pulmonary diseases with different severity levels. Images show chest radiographs (left) and corresponding saliency maps (right) for three pulmonary conditions, each with mild (row 1) and severe (row 2) cases. Red indicates positive correlation while blue indicates negative correlation with the measured parameters. **a** Chronic obstructive pulmonary disease (COPD): saliency maps of the forced expiratory volume in 1 s (FEV1) estimation. In severe cases, blue (cold) regions are more extensive and correspond to areas of increased lung transparency at the apices or throughout the lung fields, consistent with emphysematous changes. **b** Asthma: saliency maps of the FEV1 estimation. The progression from mild to severe disease shows increased blue regions concentrated around the hilar areas, suggesting involvement of central bronchi. **c** Interstitial lung disease: Saliency maps of the forced vital capacity (FVC) estimation. Severe cases demonstrate reduced red (hot) regions throughout the lung fields and increased blue regions at the lung periphery, correlating with peripheral volume loss and reticular shadowing characteristic of interstitial disease

Perhaps more intriguingly, AI has enabled novel applications that extend beyond traditional visual analysis. Recent studies have demonstrated AI’s capability to estimate cardiac and pulmonary functions directly from chest radiographs. A deep learning model for cardiac function assessment showed remarkable accuracy in estimating multiple parameters traditionally obtained through echocardiography, such as left ventricular ejection fraction (AUC 0.92) [79]. Furthermore, this model identified patients with various valvular conditions (Fig. 10) [79]. Similarly, AI-derived pulmonary function values strongly correlated with spirometry measurements (R = 0.91 for both FVC and FEV1) [80], offering an alternative assessment method for patients unable to perform conventional spirometry tests (Fig. 11).

AI-based methods are constantly redefining the limits of medical imaging. For example, AI analysis of chest radiographs has revealed unexpected potential as a biomarker of aging. A study has demonstrated strong correlations between AI-estimated and chronological age (R = 0.95) in healthy individuals [81]. Patients with known chronic conditions were found to have a larger difference between the estimated and actual age. Examples included hypertension (OR 1.04), chronic obstructive pulmonary disease (OR 1.05), and interstitial lung disease (OR 1.08) [81]. These findings suggest that chest radiographs may contain subtle indicators of biological aging and disease processes that are beyond current medical knowledge and perhaps exceed human visual capabilities.

These developments represent a paradigm shift in chest radiography, transforming this basic imaging modality into a potential source of quantitative functional and biological information. However, careful validation through larger, prospective studies remains essential to establish the clinical utility of these novel applications [82].

## MR of the lung

The Fleischner Society published a position paper in 2020, which provides consensus expert opinions regarding appropriate clinical indications for MR imaging [83–90]. There are strong data supporting the clinical use of lung MR imaging for cystic fibrosis, pulmonary hypertension evaluations, TNM staging of lung cancer and nodule characterization. There are promising data requiring further validation for pulmonary thromboembolism, pulmonary nodule detection or lung parenchyma evaluation in routine clinical practice (Table 2) [87–90].

**Table 2:** Clinical indications for lung MR imaging

Clinical indication	I	II	III
Definition	Data supporting current clinical applications	Promising data requiring further validation or regulatory approval	Investigational
Classified diseases	Cystic fibrosis	Pulmonary thromboembolism	Chronic obstructive pulmonary disease (COPD)
	Lung cancer staging	Pulmonary parenchymal abnormalities	Asthma
	Lung nodule characterization	Lung nodule detection	Interstitial lung disease
	Pulmonary hypertension	–	–

### Deep learning reconstruction

**Fig. 12:**
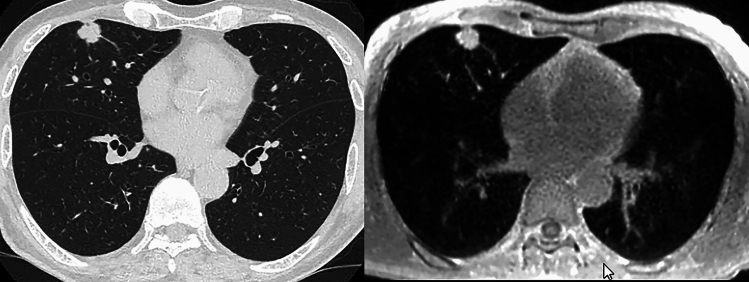
72-year old male with invasive adenocarcinoma (L to R: thin-section CT with 1 mm section thickness to UTE-MRI with same section thickness). Thin-section CT and UTE-MRI were obtained by same voxel sizes. The nodule in the right middle lobe has notch, spicula and pleural tag. Both thin-section images diagnosed this nodule as malignant and suspected as invasive adenocarcinoma. This nodule was pathologically diagnosed as invasive adenocarcinoma

Deep learning reconstruction (DLR) for MRI reconstruction aims to generate high-quality images from sampled k-space data. DL-based image reconstruction for MRI can automatically and fully exploit the available data information and recover the lost information when guided by certain prior knowledge [91–98]. Existing DLR methods can be classified into two major categories, model-based and data-driven methods. Currently, major MR vendors now provide DL- or CNN-based reconstructions for routine clinical practice (Table 3) [91–98]. With DLR for MR imaging, signal-to-noise ratio (SNR) was significantly improved without any influence on contrast-noise ratio on many conventional sequences and DWI (Fig. 12) [91–98].

**Table 3:** Commercially available and major deep learning reconstruction algorithms for MRI

Algorithm name		Algorithm developer	Algorithm class	Algorithm type	Algorithm aim
AiCE		Canon Medical Systems	DLR	Post-processing type	Denoising
AIR Recon DL		GE Healthcare	DLR	Post-processing type	Denoising & image sharpness
SmartSpeed AI		Philips Healthcare	DLR & IR (Hybrid DL-IR)	Physics-driven type	Image reconstruction & denoising
Deep Resolve	Deep Resolve Gain	Siemens Healthineers	DLR	Post-processing type	Denoising
	Deep Resolve Sharp		CNN	Post-processing type	Spatial resolution improvement
	Deep Resolve Boost		DNN	Physics-driven type	Scan time reduction
uAIFI DeepRecon		United Imaging Healthcare	CNN	Physics-driven type	Denoising & image sharpness

DLR: deep learning reconstruction, IR: iterative reconstruction, DL-IR: deep learning and iterative reconstruction, CNN: convolutional neural network, DNN: deep neural network

### Compressed sensing and other fast MR acquisition methods

Conventional reconstruction methods including Fourier transform require the scanning process to follow the Nyquist sampling theory. To obtain high-quality MR images, however, the sampling frequency should be high enough. Undersampling, on the other hand, leads to imperfect MR image reconstruction if conventional reconstruction methods are used. For this reason, it has been proposed to introduce compressed sensing (CS) for reconstructing MR images. CS accomplishes the reconstruction task mainly by exploiting the sparsity of MRI, since most MR images become sparse after being transformed into an appropriate domain, such as when total variation and wavelet transformation are used [91, 94, 96–103]. Therefore, MR images with CS and DLR can improve image quality at not only conventional, but also thin-section thicknesses [98].

### MR imaging with ultra-short echo time (UTE-MRI) less than 200 μs

**Fig. 13:**
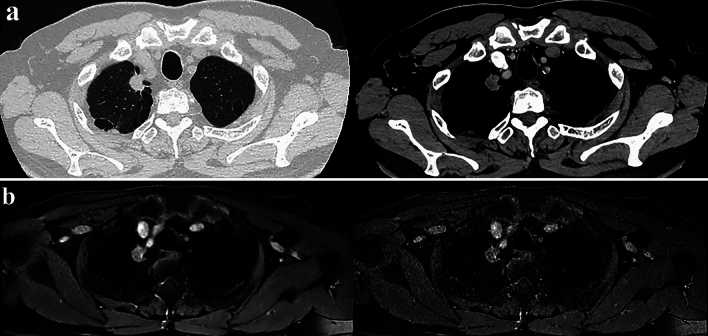
68-year old male with invasive adenocarcinoma as having mediastinal invasion. **a**: (L to R: thin-section CT with lung window setting to contrast-enhanced (CE-) thin-section CT with mediastinal window setting). All thin-section images were demonstrated as 1-mm section thickness with same voxel size. Thin-section CT with lung window setting demonstrates a nodule, whose long axis diameter was 21 mm, with notch, spicula and pleural tags. This nodule was not suspected right subclavian artery invasion on CE-thin-section CT with mediastinal window setting. On CT examination, this nodule was diagnosed as T1c. **b**: (L to R: fat-suppressed CE-3D thin-section T1-weighted images obtained by Quick 3D provided by Canon Medical Systems Corporation and reconstructed with and without deep learning reconstruction [DLR]) DLR could significantly decrease image noise and improve signal-to-noise ratio (SNR) on fat-suppressed CE-3D thin-section T1-weighted images (T1WIs) obtained by Quick 3D. Although image qualities had significant differences with both fat-suppressed CE-thin-section T1WIs, each T1WI demonstrates no mediastinal fat demonstrated as low signal intensity line or area between nodule and right subclavian artery. On each T1WI, this nodule was suspected as having mediastinal invasion and diagnosed as T3. This patient underwent surgical treatment and pathologically diagnosed as T3

Since 2016, 3D GRE with UTE of less than 200 μs (UTE-MRI) has been used for lung parenchymal abnormality or nodule detection, evaluation, and classification because this technique has the potential to improve the SNR within the lung parenchyma [83–90, 104–116]. As compared with thin-section standard- or low-dose CTs, UTE-MRI showed almost perfect or substantial agreement with thin-section standard- and low-dose CTs for radiological finding evaluations [109]. Moreover, UTE-MRI has a potential to play as substation for thin-section CT for cystic fibrosis assessments, lung cancer screening or invasiveness evaluation of lung adenocarcinoma (Fig. 13) [107–116]. Furthermore, the potential of UTE-MRI is, therefore, being tested as a substitute for CT or in a complementary role for PET fused with MR imaging (PET/MRI) [117, 118]. In addition, the potential of quantitative regional T2* measurement of the lung in terms of direct T2* decay on 3D GRE with UTEs has already been tested for the assessment of lung microstructure changes in in vitro and in vivo studies [87–90, 105, 106, 119–123]. UTE-MRI may, therefore, be considered a promising technique, although it is still warranting further investigations for routine clinical practice.

## My own reflection and score

Briefly, I graduated from Kyoto University Faculty of Medicine in 1983. My professional clinical training includes Junior Residency (Medicine, Surgery, Pediatrics, Emergency, ICU, Anesthesiology, Clinical Pathology, and Radiology: 1983–1985) and Senior Residency in Radiology (1985–1987) at Tenri Hospital in Nara, Japan, Diagnostic Radiology Residency at the Hospital of the University of Pennsylvania (1992–1995), and Clinical Fellowship in magnetic resonance imaging at Beth Israel Hospital Boston (1995–1996). My research training includes Research Fellow at the Hospital of the University of Pennsylvania in magnetic resonance imaging under Professor Herbert Y. Kressel (1987 and 1990–1991), and Graduate School of Medicine (1987–1991) at Kyoto University under Professor Junji Konishi. My past faculty appointments include at Kyoto University: Assistant Professor (1992–1998), University of Pennsylvania: Associate Professor (1999–2002), and Harvard Medical School: Instructor (1995–1996), Assistant Professor (1998–1999), Associate Professor (1999 and 2002–2013), and Professor (2013–2024). I am board certified in diagnostic radiology in both United States and Japan, fellow of American College of Radiology and Japanese College of Radiology, and a member of The Fleischner Society since 2011, a multi-disciplinary honor society for chest imaging of 85 members in the world. I am currently serving as President-elect for The Fleischner Society and will be the President in 2025–2026.

I believe my most impactful contribution to medicine to date is the first proposal of the radiologic definition of the Interstitial Lung Abnormalities (ILA), an entity which may include the early pulmonary fibrosis for the potential early intervention and prevention of developing full-brown pulmonary fibrosis. In 2009, we analyzed 2416 CT scans in smokers in COPDGene Study. In smokers, interstitial lung abnormalities (ILA) were present on one of every 12 CT scans, which were associated with reduced total lung capacity. The results were published in the New England Journal of Medicine with an editorial. I served as co-corresponding author role representing Radiology: Washko GR et al. N Engl. J Med. 2011; 364:897–906 (Citations 409: NIH-RCR, 98.6%tile) [124]. We subsequently reported the association between MUC5B promoter polymorphism and ILA: Hunninghake GM, Hatabu H, et al. N Engl. J Med. 2013; 368:2192–2200 (Citations 307: NIH-RCR, 97.9%tile) [125]. We also reported association between ILA and all-cause mortality: Putman RK, Hatabu H, et al. JAMA 2016; 315:672–681 (Citations 300: NIH-RCR, 98.7%tile) [126]. I served as Chair of the Fleischner Position Paper Writing Committee for ILA detected incidentally on CT: Hatabu H et al. Lancet Respir. Med. 2020; 8:726–737 (Citations 268: NIH-RCR, 99.5%tile) [67].

My second most impactful contribution is on MR imaging of pulmonary vasculature and perfusion as well as lung parenchyma. In 1996, we reported the first MR imaging of pulmonary perfusion: Pulmonary perfusion: qualitative assessment with dynamic-contrast-enhanced MRI using ultra-short TE and inversion recovery turbo FLASH. Hatabu H et al. Magn Reson. Med. 1996; 36:503–508. (Citations 171: NIH-RCR, 95.2%tile) [127]. For this work, I was awarded the W.S. Moore Young Investigator Award from International Society for Magnetic Resonance in Medicine in 1996. Subsequently, we reported quantitative assessment of pulmonary perfusion: Hatabu H. et al. Magn Reson. Med. 1999; 42:1033–1038. (Citations 132: NIH-RCR, 90.3%tile) [128]. We first reported application of two fundamental MR sequences now standard in chest MR protocol in the world: Hatabu et al. MR imaging of pulmonary parenchyma with a half-Fourier single-shot turbo spin-echo sequence. Eur. J Radiol. 1999; 29:152–159. (Citations 109: NIH-RCR 88.2%tile) [129] and Alsop DC, Hatabu H. et al. Multi-slice breathhold imaging of the lung with submillisecond echo time. Magn. Reason. Med. 1995; 33:678–682 (Citations 91: NIH-RCR, 87.4%tile) [130]. Recently, I served as Chair of the Fleischner Position Paper Writing Committee for Expanding Application of Pulmonary MRI in the Clinical Evaluation of Lung Disorders: Hatabu H et al. Radiology 2020; 297:286–301 (Citations 87: NIH-RCR, 97.7%tile) [87].

My third most impactful contribution is radiologic description of drug-related pneumonitis in patients receiving immune checkpoint inhibitors and molecular targeted agents. Working with Dr. Nishino M. and Hodi FS as a member of the group, we reported the first case of immune checkpoint inhibitor-related pneumonitis: Anti-PD-1-Related Pneumonitis during Cancer Immunotherapy. Nishino M. et al. N Engl. J Med 2015; 373:288–290 (Citations 294: NIH-RCR, 97.3%tile) [131]. Incidence of Programed Cell Death Inhibitor-related Pneumonitis in Patients with Advanced Lung Cancer: A Systematic Review and Meta-Analysis. Nishino M. et al. JAMA Oncol. 2016; 2:1607–1616 (Citations 508: NIH-RCR, 99.3%tile) [132]. Recently, I served as Co-Chair of the Fleischner Position Paper Writing Committee for Chest CT Diagnosis and Clinical Management of Drug-related Pneumonitis in Patients Receiving Molecular Targeted Agents and Immune Checkpoint Inhibitors, serving as the senior author: Johkoh T et al. CHEST 2021; 159:1107–1125 (Citations 43: NIH-RCR, 92.4%tile) [133]. Radiology 2021; 298:550–566 (Citations 90: NIH-RCR 89.1%tile) [134].

Working with many young fellows and junior faculties, I was fortunate to publish more than 300 original investigative publications and over 70 review articles including 40 publications with more than 100 citations. On Web of Science, 533 publications were identified with citations of 17, 618 with average citations of 33, and h-index of 69. On iCite report of RCR, 92 publications had 80%tile or higher, of which 45 publications were more than 90%tile and 19 publications were over 95%tile. After board-certified both in Japan and United States, I have keen interest in the education and professional training. From 2006 to 2015, I served as Clinical Director of MRI, and redesigned Clinical MRI operation with increase in MR fleet from 8 scanners to 15 at that period with revenue of 80 million dollars per year to 150 million dollars per year, by creating a team of physicians and technologist leaders: our MR Modernization team was awarded Partners in Excellence Award for Teamwork in 2008 and 2011 while I served as team leader. I served as Director for Residency Education and Co-Director of Radiology Residency Program in 2002–2006 at Beth Israel Deaconess Medical Center and Harvard Medical School. I was awarded the Faculty Award for Excellence in Teaching in 2003 and Outstanding Commitment and Service Award in 2006.

## Discussion and conclusion

The topics of “early pulmonary fibrosis”, “MR imaging of pulmonary vasculature, perfusion, and parenchyma”, and “drug-related pneumonitis” happen to be the clinical questions I encountered in my first two years as a resident at Tenri Hospital in Nara. In a sense, I have been addressing those three questions in the following several decades in my career. For radiologists, there is always question of “disease-oriented” versus “modality-oriented” in research. From the point of view of the potential clinical impact, “disease-oriented” approach appears superior to “modality-oriented” approach; however, radiologists are very hard to compete with clinicians in medicine and surgery for the “disease-oriented” approach except when they happen to acquire great partners in clinical medicine. On the other hand, radiologists are often given the first-hand opportunities, when the new imaging modalities emerge. Therefore, it is natural for radiologists to utilize the emerging modalities for their new investigations. Even the famous Professor Galileo Galilei could not discover what he did without “telescope”. The new modalities serve for us as new “telescopes” for medicine.

The topics contributed to this review article included “high spatial resolution and photon-counting CT”, “upright CT”, “dynamic radiography”, “AI”, and “MRI”. As we can see, it is very natural that the topics by the above young investigators are modality- or method-oriented yet addressing the important and appropriate clinical questions. The new research in radiology have been always stimulated by the emerging technology and advancement of the computer science including AI. The modality- or method-oriented approaches will remain one of the major approaches for the investigators in radiology. For taking “disease-oriented” approach, it is necessary to plan very carefully to create the research cohorts with least biases and to build a long-term collaboration with bright yet easy-to-work-with clinicians in medicine and surgery.

Occasionally at least several times a year, I will make a list of research projects on the paper or note, sometimes in the airplane. Then I will reorder the projects in the order of importance from the top to the bottom: I will work on the top third of the projects; keep middle third just in case we have time; and try not to work on the bottom third. Please keep in mind what is the strength of your institution and environment as well as your own. Please keep yourself as an active clinical radiologist.

As of December 31, 2024, I will most likely become a Professor Emeritus at Harvard Medical School: In January 2025, I plan to return to start a new chapter of life at the Department of Radiology at University of Pennsylvania as Vice Chair for Clinical Imaging Innovation and Chief of the Center for Clinical Imaging Innovation (CCII) to achieve something impactful by collaborating with multiple groups of the established investigators. The new center will serve as resource for the rising young faculty members and trainees at University of Pennsylvania and collaborators from the universe. It stopped snowing, leaving everything on the horizon in New England equally still and white.

## References

[1] Kakinuma R, Moriyama N, Muramatsu Y, Gomi S, Suzuki M, Nagasawa H, et al. Ultra-high-resolution computed tomography of the lung: image quality of a prototype scanner. PLoS ONE. 2015;10(9): e0137165. 26352144 10.1371/journal.pone.0137165 PMC4564227

[2] Tsukagoshi S, Ota T, Fujii M, Kazama M, Okumura M, Johkoh T. Improvement of spatial resolution in the longitudinal direction for isotropic imaging in helical CT. Phys Med Biol. 2007;52(3):791–801. 17228121 10.1088/0031-9155/52/3/018

[3] Yanagawa M, Hata A, Honda O, Kikuchi N, Miyata T, Uranishi A, et al. Subjective and objective comparisons of image quality between ultra-high-resolution CT and conventional area detector CT in phantoms and cadaveric human lungs. Eur Radiol. 2018;28(12):5060–8. 29845337 10.1007/s00330-018-5491-2 PMC6223853

[4] Miyata T, Yanagawa M, Hata A, Honda O, Yoshida Y, Kikuchi N, et al. Influence of field of view size on image quality: ultra-high-resolution CT vs. conventional high-resolution CT. Eur Radiol. 2020;30(6):3324–33. 32072253 10.1007/s00330-020-06704-0 PMC7248011

[5] Hata A, Yanagawa M, Honda O, Kikuchi N, Miyata T, Tsukagoshi S, et al. Effect of matrix size on the image quality of ultra-high-resolution CT of the lung: comparison of 512 x 512, 1024 x 1024, and 2048 x 2048. Acad Radiol. 2018;25(7):869–76. 29373211 10.1016/j.acra.2017.11.017

[6] Yanagawa M, Tsubamoto M, Satoh Y, Hata A, Miyata T, Yoshida Y, et al. Lung adenocarcinoma at CT with 0.25-mm section thickness and a 2048 matrix: high-spatial-resolution imaging for predicting invasiveness. Radiology. 2020;297(2):462–71. 32897161 10.1148/radiol.2020201911

[7] Ninomiya K, Yanagawa M, Tsubamoto M, Sato Y, Suzuki Y, Hata A, et al. Prediction of solid and micropapillary components in lung invasive adenocarcinoma: radiomics analysis from high-spatial-resolution CT data with 1024 matrix. Jpn J Radiol. 2024;42(6):590–8. 38413550 10.1007/s11604-024-01534-2 PMC11139717

[8] Egashira R, Jacob J, Kokosi MA, Brun AL, Rice A, Nicholson AG, et al. Diffuse pulmonary ossification in fibrosing interstitial lung diseases: prevalence and associations. Radiology. 2017;284(1):255–63. 28182861 10.1148/radiol.2017152419

[9] Hata A, Yanagawa M, Tsubamoto M, Doi S, Yoshida Y, Miyata T, et al. Detectability of pulmonary ossifications in fibrotic lung on ultra-high-resolution CT using 2048 matrix size and 0.25-mm slice thickness. Sci Rep. 2021;11(1):15119. 34302045 10.1038/s41598-021-94596-5 PMC8302596

[10] Yanagawa M, Ito R, Nozaki T, Fujioka T, Yamada A, Fujita S, et al. New trend in artificial intelligence-based assistive technology for thoracic imaging. Radiol Med. 2023;128(10):1236–49. 37639191 10.1007/s11547-023-01691-w PMC10547663

[11] Koetzier LR, Mastrodicasa D, Szczykutowicz TP, van der Werf NR, Wang AS, Sandfort V, et al. Deep learning image reconstruction for CT: technical principles and clinical prospects. Radiology. 2023;306(3): e221257. 36719287 10.1148/radiol.221257 PMC9968777

[12] Nakamoto A, Onishi H, Ota T, Honda T, Tsuboyama T, Fukui H, et al. Contrast-enhanced thin-slice abdominal CT with super-resolution deep learning reconstruction technique: evaluation of image quality and visibility of anatomical structures. Jpn J Radiol. 2024. 10.1007/s11604-024-01685-2. 39538066 10.1007/s11604-024-01685-2 PMC11868232

[13] Takafuji M, Kitagawa K, Mizutani S, Hamaguchi A, Kisou R, Iio K, et al. Super-resolution deep learning reconstruction for improved image quality of coronary CT angiography. Radiol Cardiothorac Imaging. 2023;5(4): e230085. 37693207 10.1148/ryct.230085 PMC10485715

[14] Orii M, Sone M, Osaki T, Ueyama Y, Chiba T, Sasaki T, et al. Super-resolution deep learning reconstruction at coronary computed tomography angiography to evaluate the coronary arteries and in-stent lumen: an initial experience. BMC Med Imaging. 2023;23(1):171. 37904089 10.1186/s12880-023-01139-7 PMC10617195

[15] McCollough CH, Rajendran K, Baffour FI, Diehn FE, Ferrero A, Glazebrook KN, et al. Clinical applications of photon counting detector CT. Eur Radiol. 2023;33(8):5309–20. 37020069 10.1007/s00330-023-09596-y PMC10330165

[16] Nakamura Y, Higaki T, Kondo S, Kawashita I, Takahashi I, Awai K. An introduction to photon-counting detector CT (PCD CT) for radiologists. Jpn J Radiol. 2023;41(3):266–82. 36255601 10.1007/s11604-022-01350-6 PMC9974724

[17] Hata A, Yanagawa M, Ninomiya K, Kikuchi N, Kurashige M, Masuda C, et al. Photon-counting detector CT radiological-histological correlation in cadaveric human lung nodules and airways. Invest Radiol. 2024. 10.1097/RLI.0000000000001117. 39159364 10.1097/RLI.0000000000001117

[18] McCollough CH, Rajendran K, Leng S. Standardization and quantitative imaging with photon-counting detector CT. Invest Radiol. 2023;58(7):451–8. 36728452 10.1097/RLI.0000000000000948 PMC10272018

[19] Hertel A, Tharmaseelan H, Rotkopf LT, Norenberg D, Riffel P, Nikolaou K, et al. Phantom-based radiomics feature test-retest stability analysis on photon-counting detector CT. Eur Radiol. 2023;33(7):4905–14. 36809435 10.1007/s00330-023-09460-z PMC10289937

[20] Si-Mohamed SA, Sigovan M, Hsu JC, Tatard-Leitman V, Chalabreysse L, Naha PC, et al. In vivo molecular k-edge imaging of atherosclerotic plaque using photon-counting CT. Radiology. 2021;300(1):98–107. 33944628 10.1148/radiol.2021203968 PMC8217298

[21] Gang GJ, Zbijewski W, Mahesh M, Thawait G, Packard N, Yorkston J, et al. Image quality and dose for a multisource cone-beam CT extremity scanner. Med Phys. 2018;45(1):144–55. 29121409 10.1002/mp.12659

[22] Jinzaki M, Yamada Y, Nagura T, Nakahara T, Yokoyama Y, Narita K, et al. Development of upright computed tomography with area detector for whole-body scans: phantom study, efficacy on workflow, effect of gravity on human body, and potential clinical impact. Invest Radiol. 2020;55(2):73–83. 31503082 10.1097/RLI.0000000000000603 PMC6948833

[23] Yamada Y, Yamada M, Yokoyama Y, Tanabe A, Matsuoka S, Niijima Y, et al. Differences in lung and lobe volumes between supine and standing positions scanned with conventional and newly developed 320-detector-row upright CT: intra-individual comparison. Respiration. 2020;99(7):598–605. 32640453 10.1159/000507265 PMC7490509

[24] Matsumoto S, Yamada Y, Yamada M, Chubachi S, Yokoyama Y, Matsuoka S, et al. Difference in the airway luminal area between the standing and supine positions using upright and conventional computed tomography. Clin Anat. 2021;34(8):1150–6. 34218460 10.1002/ca.23763

[25] Yamada Y, Yamada M, Chubachi S, Yokoyama Y, Matsuoka S, Tanabe A, et al. Comparison of inspiratory and expiratory lung and lobe volumes among supine, standing, and sitting positions using conventional and upright CT. Sci Rep. 2020;10(1):16203. 33004894 10.1038/s41598-020-73240-8 PMC7530723

[26] Norimatsu T, Nakahara T, Yamada Y, Yokoyama Y, Yamada M, Narita K, et al. Anatomical cardiac and electrocardiographic axes correlate in both upright and supine positions: an upright/supine CT study. Sci Rep. 2023;13(1):18170. 37875545 10.1038/s41598-023-45528-y PMC10598224

[27] Narita K, Yamada Y, Yamada M, Yokoyama Y, Nakahara T, Jinzaki M. Pelvic floor morphology in the standing position using upright computed tomography: age and sex differences. Int Urogynecol J. 2020;31(11):2387–93. 32500162 10.1007/s00192-020-04335-z

[28] Fujita N, Yagi M, Yamada Y, Yokoyama Y, Yamada M, Watanabe K, et al. Changes in the lumbar intervertebral foramen between supine and standing posture in patients with adult spinal deformity: a study with upright computed tomography. Skeletal Radiol. 2023;52(2):215–24. 36114881 10.1007/s00256-022-04185-4

[29] Kaneda K, Harato K, Oki S, Yamada Y, Nakamura M, Nagura T, et al. Increase in tibial internal rotation due to weight-bearing is a key feature to diagnose early-stage knee osteoarthritis: a study with upright computed tomography. BMC Musculoskelet Disord. 2022;23(1):253. 35291984 10.1186/s12891-022-05190-3 PMC8925230

[30] Yokoyama Y, Yamada Y, Kosugi K, Yamada M, Narita K, Nakahara T, et al. Effect of gravity on brain structure as indicated on upright computed tomography. Sci Rep. 2021;11(1):392. 33431952 10.1038/s41598-020-79695-z PMC7801697

[31] Yamada Y, Yamada M, Chubachi S, Yokoyama Y, Matsuoka S, Tanabe A, et al. Comparison of inspiratory and expiratory airway volumes and luminal areas among standing, sitting, and supine positions using upright and conventional CT. Sci Rep. 2022;12(1):21315. 36494466 10.1038/s41598-022-25865-0 PMC9734674

[32] Chubachi S, Yamada Y, Yamada M, Yokoyama Y, Tanabe A, Matsuoka S, et al. Differences in airway lumen area between supine and upright computed tomography in patients with chronic obstructive pulmonary disease. Respir Res. 2021;22(1):95. 33789651 10.1186/s12931-021-01692-1 PMC8010787

[33] Chubachi S, Okamori S, Yamada Y, Yamada M, Yokoyama Y, Niijima Y, et al. Differences in lung and lobe volumes between supine and upright computed tomography in patients with idiopathic lung fibrosis. Sci Rep. 2022;12(1):19408. 36371537 10.1038/s41598-022-24157-x PMC9653373

[34] Yamada Y, Chubachi S, Yamada M, Yokoyama Y, Tanabe A, Matsuoka S, et al. Comparison of lung, lobe, and airway volumes between supine and upright computed tomography and their correlation with pulmonary function test in patients with chronic obstructive pulmonary disease. Respiration. 2022;101(12):1110–20. 36353776 10.1159/000527067 PMC9811423

[35] Fukuoka R, Yamada Y, Kataoka M, Yokoyama Y, Yamada M, Narita K, et al. Estimating right atrial pressure using upright computed tomography in patients with heart failure. Eur Radiol. 2023;33(6):4073–81. 36576542 10.1007/s00330-022-09360-8 PMC10182146

[36] Tanaka R, Sanada S, Kobayashi T, Suzuki M, Matsui T, Matsui O. Computerized methods for determining respiratory phase on dynamic chest radiographs obtained by a dynamic flat-panel detector (FPD) system. J Digit Imaging. 2006;19(1):41–51. 15827824 10.1007/s10278-004-1045-z PMC3043950

[37] Hata A, Yamada Y, Tanaka R, Nishino M, Hida T, Hino T, et al. Dynamic chest x-ray using a flat-panel detector system: technique and applications. Korean J Radiol. 2021;22(4):634–51. 33289365 10.3348/kjr.2020.1136 PMC8005348

[38] Hino T, Hata A, Hida T, Yamada Y, Ueyama M, Araki T, et al. Projected lung areas using dynamic X-ray (DXR). Eur J Radiol Open. 2020;7: 100263. 32953949 10.1016/j.ejro.2020.100263 PMC7486627

[39] Yamada Y, Ueyama M, Abe T, Araki T, Abe T, Nishino M, et al. Time-resolved quantitative analysis of the diaphragms during tidal breathing in a standing position using dynamic chest radiography with a flat panel detector system (“dynamic x-ray phrenicography”): initial experience in 172 volunteers. Acad Radiol. 2017;24(4):393–400. 27989446 10.1016/j.acra.2016.11.014

[40] Yamada Y, Ueyama M, Abe T, Araki T, Abe T, Nishino M, et al. Difference in diaphragmatic motion during tidal breathing in a standing position between COPD patients and normal subjects: Time-resolved quantitative evaluation using dynamic chest radiography with flat panel detector system (“dynamic X-ray phrenicography”). Eur J Radiol. 2017;87:76–82. 28065378 10.1016/j.ejrad.2016.12.014

[41] Hida T, Yamada Y, Ueyama M, Araki T, Nishino M, Kurosaki A, et al. Time-resolved quantitative evaluation of diaphragmatic motion during forced breathing in a health screening cohort in a standing position: dynamic chest phrenicography. Eur J Radiol. 2019;113:59–65. 30927960 10.1016/j.ejrad.2019.01.034

[42] Hida T, Yamada Y, Ueyama M, Araki T, Nishino M, Kurosaki A, et al. Decreased and slower diaphragmatic motion during forced breathing in severe COPD patients: Time-resolved quantitative analysis using dynamic chest radiography with a flat panel detector system. Eur J Radiol. 2019;112:28–36. 30777216 10.1016/j.ejrad.2018.12.023

[43] Ohkura N, Tanaka R, Watanabe S, Hara J, Abo M, Nakade Y, et al. Chest dynamic-ventilatory digital radiography in chronic obstructive or restrictive lung disease. Int J Chron Obstruct Pulmon Dis. 2021;16:1393–9. 34040366 10.2147/COPD.S309960 PMC8140888

[44] FitzMaurice TS, McCann C, Nazareth DS, Walshaw MJ. Characterisation of hemidiaphragm dysfunction using dynamic chest radiography: a pilot study. ERJ Open Res. 2022. 10.1183/23120541.00343-2021. 35211619 10.1183/23120541.00343-2021 PMC8862633

[45] FitzMaurice TS, McCann C, Nazareth DS, McNamara PS, Walshaw MJ. Use of dynamic chest radiography to assess treatment of pulmonary exacerbations in cystic fibrosis. Radiology. 2022;303(3):675–81. 35289662 10.1148/radiol.212641

[46] Watase S, Sonoda A, Matsutani N, Muraoka S, Hanaoka J, Nitta N, et al. Evaluation of intrathoracic tracheal narrowing in patients with obstructive ventilatory impairment using dynamic chest radiography: a preliminary study. Eur J Radiol. 2020;129: 109141. 32593078 10.1016/j.ejrad.2020.109141

[47] Hino T, Tsunomori A, Fukumoto T, Hata A, Hida T, Yamada Y, et al. Projected lung area using dynamic X-ray (DXR) with a flat-panel detector system and automated tracking in patients with chronic obstructive pulmonary disease (COPD). Eur J Radiol. 2022;157: 110546. 36302331 10.1016/j.ejrad.2022.110546

[48] Hino T, Tsunomori A, Fukumoto T, Hata A, Ueyama M, Kurosaki A, et al. Vector-Field dynamic X-ray (VF-DXR) using optical flow method. Br J Radiol. 2022;95(1132):20201210. 34233474 10.1259/bjr.20201210 PMC9153721

[49] Hino T, Tsunomori A, Hata A, Hida T, Yamada Y, Ueyama M, et al. Vector-field dynamic x-ray (VF-DXR) using optical flow method in patients with chronic obstructive pulmonary disease. Eur Radiol Exp. 2022;6(1):4. 35099604 10.1186/s41747-021-00254-w PMC8802288

[50] Wada N, Tsunomori A, Kubo T, Hino T, Hata A, Yamada Y, et al. Assessment of pulmonary function in COPD patients using dynamic digital radiography: a novel approach utilizing lung signal intensity changes during forced breathing. Eur J Radiol Open. 2024;13: 100579. 39041056 10.1016/j.ejro.2024.100579 PMC11260941

[51] Yamada Y, Ueyama M, Abe T, Araki T, Abe T, Nishino M, et al. Difference in the craniocaudal gradient of the maximum pixel value change rate between chronic obstructive pulmonary disease patients and normal subjects using sub-mGy dynamic chest radiography with a flat panel detector system. Eur J Radiol. 2017;92:37–44. 28624018 10.1016/j.ejrad.2017.04.016

[52] Tanaka R, Matsumoto I, Takayama T, Ohkura N, Inoue D. Preoperative assessment of pleural adhesions in patients with lung cancer based on quantitative motion analysis with dynamic chest radiography: a retrospective study. J Appl Clin Med Phys. 2023;24(7): e14036. 37195266 10.1002/acm2.14036 PMC10338755

[53] Watanabe T, Suzuki E, Yoshii N, Tsuchida H, Yobita S, Uchiyama S, et al. Preoperative detection of pleural adhesions using dynamic chest radiography: prospective analysis. J Thorac Dis. 2023;15(3):1096–105. 37065574 10.21037/jtd-22-1226 PMC10089839

[54] Cè M, Oliva G, Rabaiotti FL, Macrì L, Zollo S, Aquila A, et al. Portable dynamic chest radiography: literature review and potential bedside applications. Med Sci. 2024;12(1):10. 10.3390/medsci12010010 PMC10885043 38390860

[55] Tanaka R. Dynamic chest radiography: flat-panel detector (FPD) based functional X-ray imaging. Radiol Phys Technol. 2016;9(2):139–53. 27294264 10.1007/s12194-016-0361-6

[56] Yamasaki Y, Hosokawa K, Kamitani T, Abe K, Sagiyama K, Hino T, et al. Diagnostic accuracy and added value of dynamic chest radiography in detecting pulmonary embolism: a retrospective study. Eur J Radiol Open. 2024;13: 100602. 39430407 10.1016/j.ejro.2024.100602 PMC11490836

[57] Yamasaki Y, Abe K, Kamitani T, Hosokawa K, Hida T, Sagiyama K, et al. Efficacy of dynamic chest radiography for chronic thromboembolic pulmonary hypertension. Radiology. 2023;306(3): e220908. 36346313 10.1148/radiol.220908

[58] Waite S, Scott J, Colombo D. Narrowing the gap: imaging disparities in radiology. Radiology. 2021;299(1):27–35. 33560191 10.1148/radiol.2021203742

[59] Liu RH, Lindeborg M, Ncogoza I, Nyiraneza SE, Barrera KJ, Shaye DA. Geospatial evaluation of radiologic access in Rwanda. Insights Imaging. 2024;15(1):105. 38589631 10.1186/s13244-024-01624-9 PMC11001820

[60] Yamasaki Y, Kamitani T, Sagiyama K, Hino T, Kisanuki M, Tabata K, et al. Dynamic chest radiography for pulmonary vascular diseases: clinical applications and correlation with other imaging modalities. Japan J Radiol. 2023. 10.1007/s11604-023-01483-2. 10.1007/s11604-023-01483-2 PMC10811043 37626168

[61] Yamasaki Y, Abe K, Hosokawa K, Kamitani T. A novel pulmonary circulation imaging using dynamic digital radiography for chronic thromboembolic pulmonary hypertension. Eur Heart J. 2020;41(26):2506. 32155252 10.1093/eurheartj/ehaa143 PMC7368460

[62] Yamasaki Y, Hosokawa K, Tsutsui H, Ishigami K. Pulmonary ventilation-perfusion mismatch demonstrated by dynamic chest radiography in giant cell arteritis. Eur Heart J. 2021;42(2):208–9. 32607531 10.1093/eurheartj/ehaa443 PMC7813626

[63] Yamasaki Y, Ishigami K. Dynamic chest radiography of pulmonary arteriovenous malformation. Radiology. 2021;300(2):285. 34003057 10.1148/radiol.2021204631

[64] Toyomura D, Yamamura K, Yamasaki Y. Dynamic digital radiography: a novel quantitative modality to assess the pulmonary blood flow. Eur Heart J. 2023;44(16):1479. 36883345 10.1093/eurheartj/ehad112

[65] Hiraiwa H, Sakamoto G, Ito R, Koyama Y, Kazama S, Kimura Y, et al. Dynamic chest radiography as a novel minimally invasive hemodynamic imaging method in patients with heart failure. Eur J Radiol. 2023;161: 110729. 36804311 10.1016/j.ejrad.2023.110729

[66] Okamoto H, Miyatake H, Kodama M, Matsubayashi J, Matsutani N, Fujino K, et al. Discriminative ability of dynamic chest radiography to identify left ventricular dysfunction. Circ J. 2023;88(1):159–67. 38030239 10.1253/circj.CJ-23-0429

[67] Hatabu H, Hunninghake GM, Richeldi L, Brown KK, Wells AU, Remy-Jardin M, et al. Interstitial lung abnormalities detected incidentally on CT: a position paper from the fleischner society. Lancet Respir Med. 2020;8(7):726–37. 32649920 10.1016/S2213-2600(20)30168-5 PMC7970441

[68] Hata A, Schiebler ML, Lynch DA, Hatabu H. Interstitial lung abnormalities: state of the art. Radiology. 2021;301(1):19–34. 34374589 10.1148/radiol.2021204367 PMC8487219

[69] Lee JE, Chae KJ, Suh YJ, Jeong WG, Lee T, Kim YH, et al. Prevalence and long-term outcomes of CT interstitial lung abnormalities in a health screening cohort. Radiology. 2023;306(2): e221172. 36219115 10.1148/radiol.221172

[70] Doyle TJ, Washko GR, Fernandez IE, Nishino M, Okajima Y, Yamashiro T, et al. Interstitial lung abnormalities and reduced exercise capacity. Am J Respir Crit Care Med. 2012;185(7):756–62. 22268134 10.1164/rccm.201109-1618OC PMC3326424

[71] Wille MM, Thomsen LH, Dirksen A, Petersen J, Pedersen JH, Shaker SB. Emphysema progression is visually detectable in low-dose CT in continuous but not in former smokers. Eur Radiol. 2014;24(11):2692–9. 25038853 10.1007/s00330-014-3294-7

[72] Chae KJ, Lim S, Seo JB, Hwang HJ, Choi H, Lynch D, et al. Interstitial lung abnormalities at CT in the Korean national lung cancer screening program: prevalence and deep learning-based texture analysis. Radiology. 2023;307(4): e222828. 37097142 10.1148/radiol.222828

[73] Hata A, Aoyagi K, Hino T, Kawagishi M, Wada N, Song J, et al. Automated interstitial lung abnormality probability prediction at CT: a stepwise machine learning approach in the boston lung cancer study. Radiology. 2024;312(3): e233435. 39225600 10.1148/radiol.233435 PMC11419784

[74] Lakhani P, Sundaram B. Deep learning at chest radiography: automated classification of pulmonary tuberculosis by using convolutional neural networks. Radiology. 2017;284(2):574–82. 28436741 10.1148/radiol.2017162326

[75] Shimazaki A, Ueda D, Choppin A, Yamamoto A, Honjo T, Shimahara Y, et al. Deep learning-based algorithm for lung cancer detection on chest radiographs using the segmentation method. Sci Rep. 2022;12(1):727. 35031654 10.1038/s41598-021-04667-w PMC8760245

[76] Ueda D, Yamamoto A, Shimazaki A, Walston SL, Matsumoto T, Izumi N, et al. Artificial intelligence-supported lung cancer detection by multi-institutional readers with multi-vendor chest radiographs: a retrospective clinical validation study. BMC Cancer. 2021;21(1):1120. 34663260 10.1186/s12885-021-08847-9 PMC8524996

[77] Gavelli G, Giampalma E. Sensitivity and specificity of chest x-ray screening for lung cancer. Cancer. 2000;89(S11):2453–6. 11147625 10.1002/1097-0142(20001201)89:11+<2453::aid-cncr21>3.3.co;2-d

[78] Quekel LG, Kessels AG, Goei R, van Engelshoven JM. Miss rate of lung cancer on the chest radiograph in clinical practice. Chest. 1999;115(3):720–4. 10084482 10.1378/chest.115.3.720

[79] Ueda D, Matsumoto T, Ehara S, Yamamoto A, Walston SL, Ito A, et al. Artificial intelligence-based model to classify cardiac functions from chest radiographs: a multi-institutional, retrospective model development and validation study. Lancet Digit Health. 2023;5(8):e525–33. 37422342 10.1016/S2589-7500(23)00107-3

[80] Ueda D, Matsumoto T, Yamamoto A, Walston SL, Mitsuyama Y, Takita H, et al. A deep learning-based model to estimate pulmonary function from chest x-rays: multi-institutional model development and validation study in Japan. Lancet Digit Health. 2024;6(8):e580–8. 38981834 10.1016/S2589-7500(24)00113-4

[81] Mitsuyama Y, Matsumoto T, Tatekawa H, Walston SL, Kimura T, Yamamoto A, et al. Chest radiography as a biomarker of ageing: artificial intelligence-based, multi-institutional model development and validation in Japan. Lancet Healthy Longev. 2023;4(9):e478–86. 37597530 10.1016/S2666-7568(23)00133-2

[82] Ueda D, Kakinuma T, Fujita S, Kamagata K, Fushimi Y, Ito R, et al. Fairness of artificial intelligence in healthcare: review and recommendations. Jpn J Radiol. 2024;42(1):3–15. 37540463 10.1007/s11604-023-01474-3 PMC10764412

[83] Webb WR, Gatsonis C, Zerhouni EA, Heelan RT, Glazer GM, Francis IR, et al. CT and MR imaging in staging non-small cell bronchogenic carcinoma: report of the radiologic diagnostic oncology group. Radiology. 1991;178(3):705–13. 1847239 10.1148/radiology.178.3.1847239

[84] Mayo JR, MacKay A, Muller NL. MR imaging of the lungs: value of short TE spin-echo pulse sequences. AJR Am J Roentgenol. 1992;159(5):951–6. 1414805 10.2214/ajr.159.5.1414805

[85] Hatabu H, Alsop DC, Listerud J, Bonnet M, Gefter WB. T2* and proton density measurement of normal human lung parenchyma using submillisecond echo time gradient echo magnetic resonance imaging. Eur J Radiol. 1999;29(3):245–52. 10399610 10.1016/s0720-048x(98)00169-7

[86] Ohno Y, Oshio K, Uematsu H, Nakatsu M, Gefter WB, Hatabu H. Single-shot half-Fourier RARE sequence with ultra-short inter-echo spacing for lung imaging. J Magn Reson Imaging. 2004;20(2):336–9. 15269963 10.1002/jmri.20107

[87] Hatabu H, Ohno Y, Gefter WB, Parraga G, Madore B, Lee KS, et al. Expanding applications of pulmonary MRI in the clinical evaluation of lung disorders: fleischner society position paper. Radiology. 2020;297(2):286–301. 32870136 10.1148/radiol.2020201138

[88] Schiebler ML, Parraga G, Gefter WB, Madore B, Lee KS, Ohno Y, et al. Synopsis from expanding applications of pulmonary MRI in the clinical evaluation of lung disorders: fleischner society position paper. Chest. 2021;159(2):492–5. 32941864 10.1016/j.chest.2020.09.075

[89] Tanaka Y, Ohno Y, Hanamatsu S, Obama Y, Ueda T, Ikeda H, et al. State-of-the-art MR imaging for thoracic diseases. Magn Reson Med Sci. 2022;21(1):212–34. 33952785 10.2463/mrms.rev.2020-0184 PMC9199970

[90] Ohno Y, Ozawa Y, Nagata H, Ueda T, Yoshikawa T, Takenaka D, et al. Lung magnetic resonance imaging: technical advancements and clinical applications. Invest Radiol. 2024;59(1):38–52. 37707840 10.1097/RLI.0000000000001017

[91] Ueda T, Ohno Y, Yamamoto K, Iwase A, Fukuba T, Hanamatsu S, et al. Compressed sensing and deep learning reconstruction for women’s pelvic MRI denoising: utility for improving image quality and examination time in routine clinical practice. Eur J Radiol. 2021;134: 109430. 33276249 10.1016/j.ejrad.2020.109430

[92] Ueda T, Ohno Y, Yamamoto K, Murayama K, Ikedo M, Yui M, et al. Deep learning reconstruction of diffusion-weighted mri improves image quality for prostatic imaging. Radiology. 2022;303(2):373–81. 35103536 10.1148/radiol.204097

[93] Wang S, Cao G, Wang Y, Liao S, Wang Q, Shi J, et al. Review and prospect: artificial intelligence in advanced medical imaging. Front Radiol. 2021;1: 781868. 37492170 10.3389/fradi.2021.781868 PMC10365109

[94] Obama Y, Ohno Y, Yamamoto K, Ikedo M, Yui M, Hanamatsu S, et al. MR imaging for shoulder diseases: effect of compressed sensing and deep learning reconstruction on examination time and imaging quality compared with that of parallel imaging. Magn Reson Imaging. 2022;94:56–63. 35934207 10.1016/j.mri.2022.08.004

[95] Hanamatsu S, Murayama K, Ohno Y, Yamamoto K, Yui M, Toyama H. Deep learning reconstruction for brain diffusion-weighted imaging: efficacy for image quality improvement, apparent diffusion coefficient assessment, and intravoxel incoherent motion evaluation in in vitro and in vivo studies. Diagn Interv Radiol. 2023;29(5):664–73. 37554957 10.4274/dir.2023.232149 PMC10679550

[96] Nagata H, Ohno Y, Yoshikawa T, Yamamoto K, Shinohara M, Ikedo M, et al. Compressed sensing with deep learning reconstruction: Improving capability of gadolinium-EOB-enhanced 3D T1WI. Magn Reson Imaging. 2024;108:67–76. 38309378 10.1016/j.mri.2024.01.015

[97] Ueda T, Yamamoto K, Yazawa N, Tozawa I, Ikedo M, Yui M, et al. Efficacy of compressed sensing and deep learning reconstruction for adult female pelvic MRI at 1.5 T. Eur Radiol Exp. 2024;8(1):103. 39254920 10.1186/s41747-024-00506-5 PMC11387279

[98] Takenaka D, Ozawa Y, Yamamoto K, Shinohara M, Ikedo M, Yui M, et al. Deep learning reconstruction to improve the quality of MR imaging: evaluating the best sequence for T-category assessment in non-small cell lung cancer patients. Magn Reson Med Sci. 2024;23(4):487–501. 37661425 10.2463/mrms.mp.2023-0068 PMC11447466

[99] Lustig M, Donoho D, Pauly JM. Sparse MRI: the application of compressed sensing for rapid MR imaging. Magn Reson Med. 2007;58(6):1182–95. 17969013 10.1002/mrm.21391

[100] Liang D, Liu B, Wang J, Ying L. Accelerating SENSE using compressed sensing. Magn Reson Med. 2009;62(6):1574–84. 19785017 10.1002/mrm.22161

[101] Qu X, Guo D, Ning B, Hou Y, Lin Y, Cai S, et al. Undersampled MRI reconstruction with patch-based directional wavelets. Magn Reson Imaging. 2012;30(7):964–77. 22504040 10.1016/j.mri.2012.02.019

[102] Ikeda H, Ohno Y, Murayama K, Yamamoto K, Iwase A, Fukuba T, et al. Compressed sensing and parallel imaging accelerated T2 FSE sequence for head and neck MR imaging: comparison of its utility in routine clinical practice. Eur J Radiol. 2021;135: 109501. 33395594 10.1016/j.ejrad.2020.109501

[103] Matsuyama T, Ohno Y, Yamamoto K, Ikedo M, Yui M, Furuta M, et al. Comparison of utility of deep learning reconstruction on 3D MRCPs obtained with three different k-space data acquisitions in patients with IPMN. Eur Radiol. 2022;32(10):6658–67. 35687136 10.1007/s00330-022-08877-2

[104] Ohno Y, Ozawa Y, Koyama H, Yoshikawa T, Takenaka D, Nagata H, et al. State of the Art MR imaging for lung cancer TNM stage evaluation. Cancers (Basel). 2023. 10.3390/cancers15030950. 36765907 10.3390/cancers15030950 PMC9913625

[105] Ohno Y, Seo JB, Parraga G, Lee KS, Gefter WB, Fain SB, et al. Pulmonary functional imaging: part 1-state-of-the-art technical and physiologic underpinnings. Radiology. 2021;299(3):508–23. 33825513 10.1148/radiol.2021203711 PMC8165947

[106] Gefter WB, Lee KS, Schiebler ML, Parraga G, Seo JB, Ohno Y, et al. Pulmonary functional imaging: part 2-state-of-the-art clinical applications and opportunities for improved patient care. Radiology. 2021;299(3):524–38. 33847518 10.1148/radiol.2021204033 PMC8165948

[107] Johnson KM, Fain SB, Schiebler ML, Nagle S. Optimized 3D ultrashort echo time pulmonary MRI. Magn Reson Med. 2013;70(5):1241–50. 23213020 10.1002/mrm.24570 PMC4199575

[108] Gibiino F, Sacolick L, Menini A, Landini L, Wiesinger F. Free-breathing, zero-TE MR lung imaging. MAGMA. 2015;28(3):207–15. 25200814 10.1007/s10334-014-0459-y

[109] Ohno Y, Koyama H, Yoshikawa T, Seki S, Takenaka D, Yui M, et al. Pulmonary high-resolution ultrashort TE MR imaging: comparison with thin-section standard- and low-dose computed tomography for the assessment of pulmonary parenchyma diseases. J Magn Reson Imaging. 2016;43(2):512–32. 26223818 10.1002/jmri.25008

[110] Dournes G, Menut F, Macey J, Fayon M, Chateil JF, Salel M, et al. Lung morphology assessment of cystic fibrosis using MRI with ultra-short echo time at submillimeter spatial resolution. Eur Radiol. 2016;26(11):3811–20. 26843010 10.1007/s00330-016-4218-5

[111] Ohno Y, Koyama H, Yoshikawa T, Kishida Y, Seki S, Takenaka D, et al. Standard-, reduced-, and no-dose thin-section radiologic examinations: comparison of capability for nodule detection and nodule type assessment in patients suspected of having pulmonary nodules. Radiology. 2017;284(2):562–73. 28263700 10.1148/radiol.2017161037

[112] Ohno Y, Takenaka D, Yoshikawa T, Yui M, Koyama H, Yamamoto K, et al. Efficacy of ultrashort echo time pulmonary mri for lung nodule detection and lung-RADS classification. Radiology. 2022;302(3):697–706. 34846203 10.1148/radiol.211254

[113] Wielputz MO, Lee HY, Koyama H, Yoshikawa T, Seki S, Kishida Y, et al. Morphologic characterization of pulmonary nodules with ultrashort TE MRI at 3T. AJR Am J Roentgenol. 2018;210(6):1216–25. 29547055 10.2214/AJR.17.18961

[114] Wielputz MO, Triphan SMF, Ohno Y, Jobst BJ, Kauczor HU. Outracing lung signal decay - potential of ultrashort echo time MRI. Rofo. 2019;191(5):415–23. 30257269 10.1055/a-0715-2246

[115] Serai SD, Rapp JB, States LJ, Andronikou S, Ciet P, Lee EY. Pediatric lung MRI: currently available and emerging techniques. AJR Am J Roentgenol. 2021;216(3):781–90. 33474982 10.2214/AJR.20.23104

[116] Ohno Y, Yui M, Yamamoto K, Ikedo M, Oshima Y, Hamabuchi N, et al. Pulmonary MRI with ultra-short TE using single- and dual-echo methods: comparison of capability for quantitative differentiation of non- or minimally invasive adenocarcinomas from other lung cancers with that of standard-dose thin-section CT. Eur Radiol. 2024;34(2):1065–76. 37580601 10.1007/s00330-023-10105-4

[117] Burris NS, Johnson KM, Larson PE, Hope MD, Nagle SK, Behr SC, et al. Detection of small pulmonary nodules with ultrashort echo time sequences in oncology patients by using a PET/MR system. Radiology. 2016;278(1):239–46. 26133050 10.1148/radiol.2015150489 PMC4699498

[118] Zeng F, Nogami M, Ueno YR, Kanda T, Sofue K, Kubo K, et al. Diagnostic performance of zero-TE lung MR imaging in FDG PET/MRI for pulmonary malignancies. Eur Radiol. 2020;30(9):4995–5003. 32300969 10.1007/s00330-020-06848-z PMC7431435

[119] Takahashi M, Togao O, Obara M, van Cauteren M, Ohno Y, Doi S, et al. Ultra-short echo time (UTE) MR imaging of the lung: comparison between normal and emphysematous lungs in mutant mice. J Magn Reson Imaging. 2010;32(2):326–33. 20677258 10.1002/jmri.22267 PMC2915456

[120] Togao O, Tsuji R, Ohno Y, Dimitrov I, Takahashi M. Ultrashort echo time (UTE) MRI of the lung: assessment of tissue density in the lung parenchyma. Magn Reson Med. 2010;64(5):1491–8. 20574988 10.1002/mrm.22521

[121] Ohno Y, Koyama H, Yoshikawa T, Matsumoto K, Takahashi M, Van Cauteren M, et al. T2* measurements of 3-T MRI with ultrashort TEs: capabilities of pulmonary function assessment and clinical stage classification in smokers. AJR Am J Roentgenol. 2011;197(2):W279–85. 21785054 10.2214/AJR.10.5350

[122] Ohno Y, Nishio M, Koyama H, Takenaka D, Takahashi M, Yoshikawa T, et al. Pulmonary MR imaging with ultra-short TEs: utility for disease severity assessment of connective tissue disease patients. Eur J Radiol. 2013;82(8):1359–65. 23523024 10.1016/j.ejrad.2013.02.031

[123] Ohno Y, Nishio M, Koyama H, Yoshikawa T, Matsumoto S, Seki S, et al. Pulmonary 3 T MRI with ultrashort TEs: influence of ultrashort echo time interval on pulmonary functional and clinical stage assessments of smokers. J Magn Reson Imaging. 2014;39(4):988–97. 24123342 10.1002/jmri.24232

[124] Washko GR, Hunninghake GM, Fernandez IE, Nishino M, Okajima Y, Yamashiro T, et al. Lung volumes and emphysema in smokers with interstitial lung abnormalities. N Engl J Med. 2011;364(10):897–906. 21388308 10.1056/NEJMoa1007285 PMC3074462

[125] Hunninghake GM, Hatabu H, Okajima Y, Gao W, Dupuis J, Latourelle JC, et al. MUC5B promoter polymorphism and interstitial lung abnormalities. N Engl J Med. 2013;368(23):2192–200. 23692170 10.1056/NEJMoa1216076 PMC3747636

[126] Putman RK, Hatabu H, Araki T, Gudmundsson G, Gao W, Nishino M, et al. Association between interstitial lung abnormalities and all-cause mortality. JAMA. 2016;315(7):672–81. 26881370 10.1001/jama.2016.0518 PMC4828973

[127] Hatabu H, Gaa J, Kim D, Li W, Prasad PV, Edelman RR. Pulmonary perfusion: qualitative assessment with dynamic contrast-enhanced MRI using ultra-short TE and inversion recovery turbo FLASH. Magn Reson Med. 1996;36(4):503–8. 8892200 10.1002/mrm.1910360402

[128] Hatabu H, Tadamura E, Levin DL, Chen Q, Li W, Kim D, et al. Quantitative assessment of pulmonary perfusion with dynamic contrast-enhanced MRI. Magn Reson Med. 1999;42(6):1033–8. 10571924 10.1002/(sici)1522-2594(199912)42:6<1033::aid-mrm7>3.0.co;2-7

[129] Hatabu H, Gaa J, Tadamura E, Edinburgh KJ, Stock KW, Garpestad E, et al. MR imaging of pulmonary parenchyma with a half-Fourier single-shot turbo spin-echo (HASTE) sequence. Eur J Radiol. 1999;29(2):152–9. 10374663 10.1016/s0720-048x(98)00167-3

[130] Alsop DC, Hatabu H, Bonnet M, Listerud J, Gefter W. Multi-slice, breathhold imaging of the lung with submillisecond echo times. Magn Reson Med. 1995;33(5):678–82. 7596272 10.1002/mrm.1910330513

[131] Nishino M, Sholl LM, Hodi FS, Hatabu H, Ramaiya NH. Anti-PD-1-related pneumonitis during cancer immunotherapy. N Engl J Med. 2015;373(3):288–90. 26176400 10.1056/NEJMc1505197 PMC4539956

[132] Nishino M, Giobbie-Hurder A, Hatabu H, Ramaiya NH, Hodi FS. Incidence of programmed cell death 1 inhibitor-related pneumonitis in patients with advanced cancer: a systematic review and meta-analysis. JAMA Oncol. 2016;2(12):1607–16. 27540850 10.1001/jamaoncol.2016.2453

[133] Johkoh T, Lee KS, Nishino M, Travis WD, Ryu JH, Lee HY, et al. Chest CT diagnosis and clinical management of drug-related pneumonitis in patients receiving molecular targeting agents and immune checkpoint inhibitors: a position paper from the fleischner society. Chest. 2021;159(3):1107–25. 33450293 10.1016/j.chest.2020.11.027

[134] Johkoh T, Lee KS, Nishino M, Travis WD, Ryu JH, Lee HY, et al. Chest CT diagnosis and clinical management of drug-related pneumonitis in patients receiving molecular targeting agents and immune checkpoint inhibitors: a position paper from the fleischner society. Radiology. 2021;298(3):550–66. 33434111 10.1148/radiol.2021203427

